# Identification of immune-related candidate biomarkers in plasma of patients with sporadic vestibular schwannoma

**DOI:** 10.1126/sciadv.adf7295

**Published:** 2023-11-10

**Authors:** Sasa Vasilijic, Nadia A. Atai, Hiroshi Hyakusoku, Steven Worthington, Yin Ren, Jessica E. Sagers, Mehmet I. Sahin, Alyssa Brown, Rohan Reddy, Charvi Malhotra, Takeshi Fujita, Lukas D. Landegger, Richard Lewis, D. Bradley Welling, Konstantina M. Stankovic

**Affiliations:** ^1^ Department of Otolaryngology–Head and Neck Surgery, Stanford University School of Medicine, Stanford, CA, USA.; ^2^ Department of Otolaryngology–Head and Neck Surgery, Massachusetts Eye and Ear and Harvard Medical School, Boston, MA, USA.; ^3^ Department of Otorhinolaryngology, Yokosuka Kyosai Hospital, Kanagawa, Japan.; ^4^ Harvard Institute for Quantitative Social Science, Harvard University, Cambridge, MA, USA.; ^5^ Department of Neurology, Harvard Medical School, Boston, MA, USA.; ^6^ Department of Neurosurgery, Stanford University School of Medicine, Stanford, CA, USA.; ^7^ Wu Tsai Neurosciences Institute, Stanford University, Stanford, CA, USA.

## Abstract

Vestibular schwannoma (VS) is an intracranial tumor arising from neoplastic Schwann cells and typically presenting with hearing loss. The traditional belief that hearing deficit is caused by physical expansion of the VS, compressing the auditory nerve, does not explain the common clinical finding that patients with small tumors can have profound hearing loss, suggesting that tumor-secreted factors could influence hearing ability in VS patients. We conducted profiling of patients’ plasma for 66 immune-related factors in patients with sporadic VS (*N* > 170) and identified and validated candidate biomarkers associated with tumor size (S100B) and hearing (MCP-3). We further identified a nine-biomarker panel (TNR-R2, MIF, CD30, MCP-3, IL-2R, BLC, TWEAK, eotaxin, and S100B) with outstanding discriminatory ability for VS. These findings revealed possible therapeutic targets for VS, providing a unique diagnostic tool that may predict hearing change and tumor growth in VS patients, and may inform the timing of tumor resection to preserve hearing.

## Teaser

Immune profiling of plasma of patients with vestibular schwannoma reveals biomarkers correlating with hearing loss and tumor size.

## INTRODUCTION

A vestibular schwannoma (VS) is an intracranial tumor arising from neoplastic Schwann cells in the vestibular branch of the eighth cranial nerve (*1*). VS is primarily caused by mutations in the neurofibromin 2 (*NF2*) gene disrupting production of merlin, a tumor suppressor. *NF2* mutations may be tumor specific, as in unilateral, sporadic VS, or rare germline mutations leading to bilateral VS and *NF2*-related schwannomatosis, formerly known as neurofibromatosis type 2 (NF2) (U.S. incidence 1:10,000 versus 1:33,000, respectively) (*2*–*4*). The most common symptoms of VS are related to disturbed cochlear nerve function (95%) and manifest as sensorineural hearing loss (*5*), tinnitus (60% of patients), followed by balance dysfunction (*5*, *6*). Although histologically nonmalignant, VS can cause substantial morbidity due to where it typically arises within the internal auditory canal, and can be life-threatening if unchecked expansion into the cerebellopontine angle compresses the brainstem (*7*).

VS management is informed by the rate of tumor growth, which is monitored with clinical imaging [i.e., magnetic resonance imaging (MRI)] (*8*). Growing tumors are typically treated with microsurgical resection or stereotactic radiation therapy. However, the size and location of sporadic VS do not necessarily correlate with the severity of the tumor-associated hearing loss (*9*); thus, the “wait and scan strategy” may be insufficient to determine the ideal timing for tumor resection to prevent progressive hearing loss (*10*–*12*).

The identification of blood biomarkers whose levels correlate with hearing loss severity or tumor size, or are prognostic of clinical outcomes, could have immense value for the management of VS (*13*). Prior attempts to characterize potential blood biomarkers have focused on *NF2*-related schwannomatosis rather than the more common sporadic VS, which comprises >90% of cases (*2*, *3*) and had limited sample sizes (<30 patients) (*14*–*16*). Therefore, we conducted extensive immune profiling of blood plasma to identify candidate biomarkers of the tumor and related hearing loss in sporadic VS, informed by our previous studies in vitro or among smaller cohorts. For example, matrix metalloproteinase-14 (MMP-14) is the most abundant MMP in VS, with differential expression between tumors associated with poor hearing (PH) versus good hearing (GH) (*17*). Additionally, sporadic VSs associated with GH secreted high levels of fibroblast growth factor 2 (FGF-2), which had an otoprotective effect in vitro (*18*). Conversely, higher secreted levels of tumor necrosis factor–α (TNF-α) were associated with worse VS-induced hearing loss and had an ototoxic effect in vitro (*19*). Finally, interleukin-18 (IL-18) levels were significantly elevated in the tumors of VS-PH patients compared to VS-GH patients (*20*).

Motivated by these data, we quantified the levels of FGF-2, IL-18, and TNF-α in the plasma of >170 patients with sporadic VS and extended the analysis to 66 cytokines, chemokines, growth factors, and cell surface proteins (table S1). We identified and analyzed candidate biomarkers with differential concentrations in the plasma of patients with and without VS (controls) and examined their association with preoperative hearing and tumor volume. Finally, we assessed the diagnostic utility of the candidate biomarkers and a composite biomarker panel in discriminating between VS patients and controls.

## RESULTS

### Patient characteristics

**Fig. 1.**
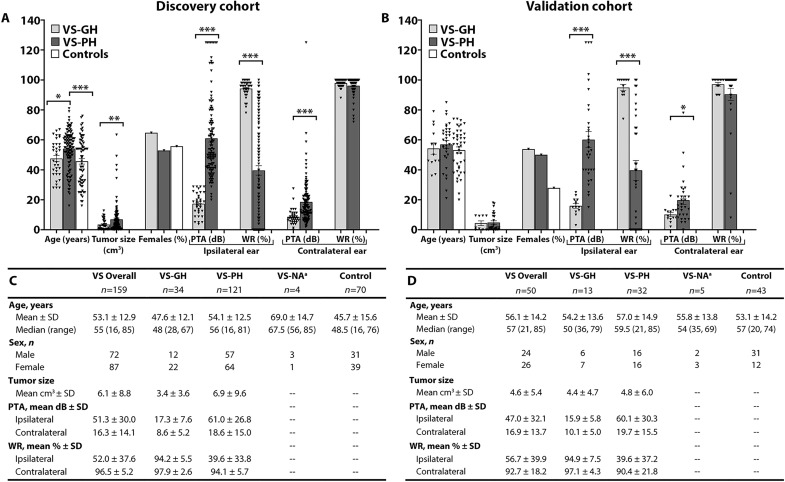
Demographic and clinical characteristics of the VS patients and controls. (**A** and **C**) Discovery cohort; (**B** and **D**) Validation cohort; NA, not available; PTA, pure-tone average; VS, vestibular schwannoma; VS-GH, VS patients with good hearing; VS-PH, VS patients with poor hearing; WR, word recognition. *P < 0.05,**P < 0.01, ***P < 0.001. Note: ^a^VS-NA refers to the small group of VS-PH patients in the discovery cohort who did not have quantitative audiograms available (*n* = 4).

A total of 159 patients with sporadic VS, including 34 with GH and 121 with PH (fig. S1A), were included for comparison with 70 controls. In this discovery cohort, VS patients and controls had similar mean ages (53 versus 46 years) and proportion of females (55 to 56%); VS-PH patients were significantly older than controls (53 years; *P* = 0.002) (Fig. 1A). Compared with VS-GH patients, VS-PH patients had significantly larger tumor volume (6.9 versus 3.4 cm^3^, *P* = 0.009), worse ipsilateral pure-tone average (PTA; 61.0 versus 17.3 dB) and word recognition percentage (WR; 39.6% versus 94.2%), and worse contralateral PTA (18.6 versus 8.6 dB; all *P* < 0.005) (fig. S2A).

In the validation cohort, a total of 50 patients with sporadic VS were studied, including 13 with GH and 32 with PH (fig. S1B), and compared to 43 controls. VS patients and controls had similar mean ages (56 versus 53 years) but not proportion of females (52% versus 28%) (Fig. 1B). Compared with VS-GH patient, VS-PH patients had significantly worse ipsilateral PTA (60.1 versus 15.9 dB) and WR score (39.6% versus 94.9%; all *P* < 0.005), and worse contralateral PTA (19.7 versus 10.1 dB; all *P* = 0.015) (fig. S2B). Worse hearing in contralateral ears of VS-PH patients is consistent with a previous report (*21*) and attributed to tumor-secreted ototoxic factors that may percolate through the cerebrospinal fluid of blood to reach the contralateral ear and affect its hearing.

### Plasma levels of candidate biomarkers

#### VS patients versus controls

**Fig. 2.**
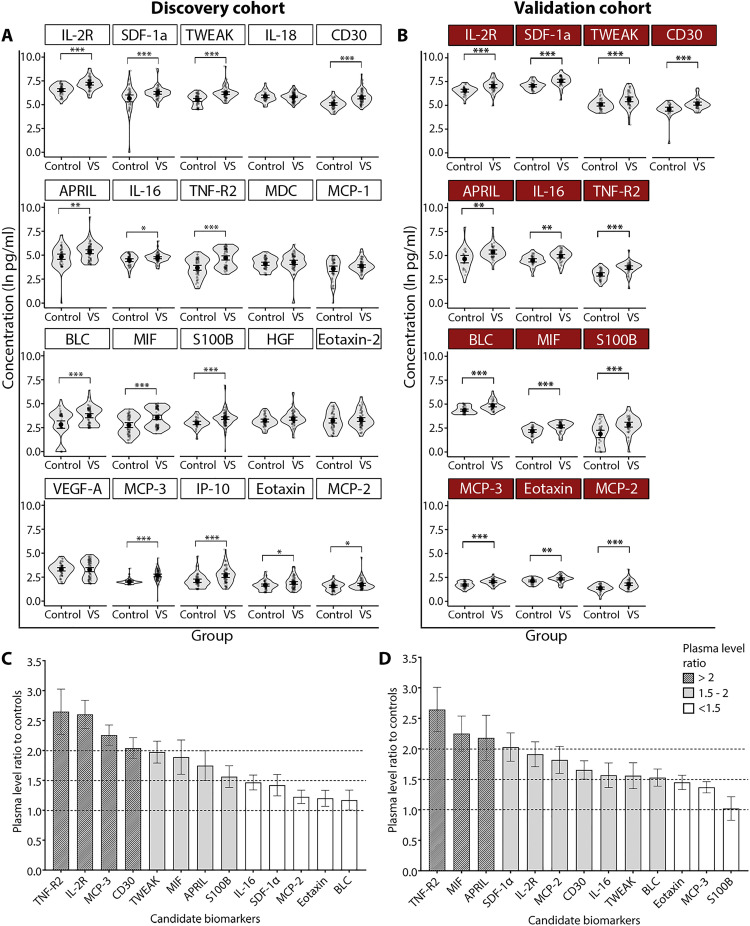
Comparison of candidate biomarker plasma levels between VS patients and controls in the discovery and validation cohorts. (**A**) The concentrations of 14 candidate biomarkers significantly differed between VS patients and controls (discovery cohort). (**B**) Thirteen of 14 candidate biomarkers were confirmed significantly different in the validation cohort. *P_adj_ < 0.05, **P_adj_ < 0.01, ***P_adj_ < 0.001. Ratios of candidate biomarker levels between VS patients and controls in the discovery (**C**) and validation (**D**) cohorts. The full names of candidate biomarkers are listed in table S1. *P*_adj_, adjusted *P* value corrected for multiple comparisons.

Twenty of the 66 profiled factors were detectable in the plasma of >75% of VS patients and considered candidate biomarkers (table S2). In both VS and control patients, the highest mean plasma values were for IL-2R, SDF-1α, and TWEAK, while the lowest values were for MCP-2, eotaxin, and MCP-3. The levels of candidate biomarkers measured in controls are in agreement with previous reports (*22*–*26*). The levels of 14 candidate biomarkers significantly differed between the VS and control groups in discovery cohort (Fig. 2A), and 13 of them were confirmed as significantly different in validation cohort (Fig. 2B). The most elevated factor among VS patients versus controls was TNF-R2 when adjusting for multiple comparisons (*P*_adj_ < 0.001), where the ratio of plasma levels was ~2.5 times higher in both cohorts (Fig. 2, C and D). TNF-α and FGF-2 were detected at very low levels in only 24% and 43% of analyzed patients, respectively, and were omitted from the analysis (table S2).

Eight validated biomarkers (MCP-3, CD30, IL-2R, TWEAK, TNF-R2, S100B, MIF, and BLC) were significantly elevated among VS patients of both sexes compared to controls (all *P*_adj_ < 0.05) (fig. S3). Eotaxin, MCP-2, and SDF-1α were significantly elevated only in male VS patients, while APRIL was only elevated in female VS patients (all *P*_adj_ < 0.05).

#### VS-GH and VS-PH patients versus controls

Compared to controls, 10 candidate biomarkers (MCP-3, CD30, S100B, TNF-R2, TWEAK, IL-2R, MIF, BLC, IL-16, and SDF-1α) were significantly elevated in both VS-GH and VS-PH patients, while APRIL and MCP-1 significantly differed only in VS-PH patients (fig. S4). Controlling for sex and age, VS-GH patients had the highest increases of MCP-3 (45.10%) and BLC (39.50%).

Compared to controls, there were significant sex simple effects among VS-PH patients of both sexes for levels of CD30, IL-2R, MCP-3, MIF, S100B, TNF-R2, and TWEAK, and among males only for BLC, eotaxin, MCP-1, MCP-2, and SDF-1α (*P*_adj_ < 0.05) (fig. S5A). There were significant sex simple effects among VS-GH patients of both sexes versus controls for IL-2R and MCP-3; among females only for CD30, S100B, and TWEAK; and among males only for MIF, SDF-1α, and TNF-R2 (all *P*_adj_ < 0.05) (fig. S5B).

#### VS-GH versus VS-PH patients

The level of IL-16 was significantly higher in VS-GH versus VS-PH patients, and MCP-3 had the highest significant percent change between groups when controlling for sex, age, and tumor volume (both *P*_adj_ < 0.05) (fig. S4).

### Interaction of biomarker levels with preoperative hearing

**Fig. 3.**
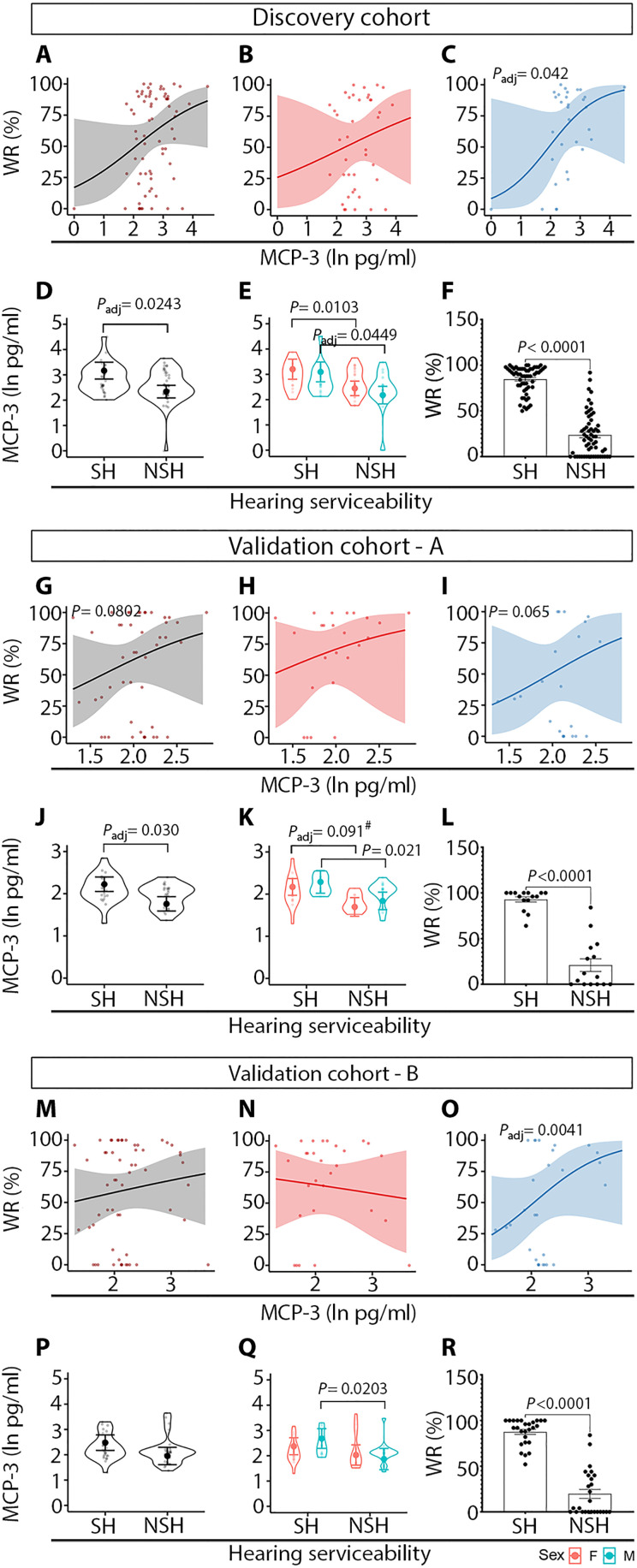
MCP-3 is associated with preoperative word recognition score and is elevated in VS patients with SH. The discovery cohort (**A** to **F**) and validation cohort-A (**G** to **L**) were composed of 78 and 50 VS patients, respectively. Validation cohort-B (**M** to **R**) was created by including additional 13 randomly selected patients out of 78 VS patients from the discovery cohort to reach a balanced number of patients in both groups. SH was defined as AAO-HNS class A and B hearing (PTA ≤ 50 dB and WR score ≥ 50%). NSH was defined as AAO-HNS class C and D hearing (either PTA > 50 dB or WRS < 50%). AAO-HNS, American Academy of Otolaryngology–Head and Neck Surgery; FDR, false discovery rate; #, statistically significant at the FDR of 0.10. Gray, patients of both sexes; red, female sex; blue, male sex.

The relationships between candidate biomarker levels and preoperative PTA and WR were assessed among VS patients with a robust linear model (RLM) and a fractional logit model (FLM), respectively. There were no significant associations with PTA and biomarker levels, or sex-specific effects. However, there was a significant association between ipsilateral WR scores and MCP-3 levels (*P*_adj_ = 0.042) in the discovery cohort of male VS patients (Fig. 3C relative to Fig. 3, A and B). According to the odds ratio, a 1-unit increase in MCP-3 natural log was associated with a WR increase of 135.5%. Furthermore, MCP-3 levels were significantly elevated in patients with serviceable hearing (SH) (*P*_adj_ = 0.0243), defined as PTA ≤ 50 dB and WR score ≥ 50% (Fig. 3D), and male VS patients (*P*_adj_ = 0.0243) (Fig. 3E) whose WR scores were significantly higher compared to patients with non-SH (NSH) (Fig. 3F). In the validation cohort, the association between MCP-3 levels with WR scores showed the same trend as in the discovery cohort (Fig. 3, G to I) and approached the criterion for significance (*P* = 0.065 in male VS patients) (Fig. 3I). The significantly elevated levels of MCP-3 in the patients with SH were confirmed in the validation cohort (Fig. 3, J and K). To test whether the trend in WR score and MCP-3 levels observed in the validation cohort (Fig. 3I) would reach statistical significance by increasing sample size, the validation cohort (validation-A) was augmented by adding 13 randomly selected patients out of 78 VS patients from the discovery cohort to reach a balanced number of patients in both groups [65 and 63 patients in the discovery and balanced validation (validation-B) cohorts, respectively]. This analysis validated a significant association between WR score and MCP-3 levels in male VS patients (*P*_adj_ = 0.0041) (Fig. 3O).

### Interaction of biomarker levels with preoperative tumor volume

**Fig. 4.**
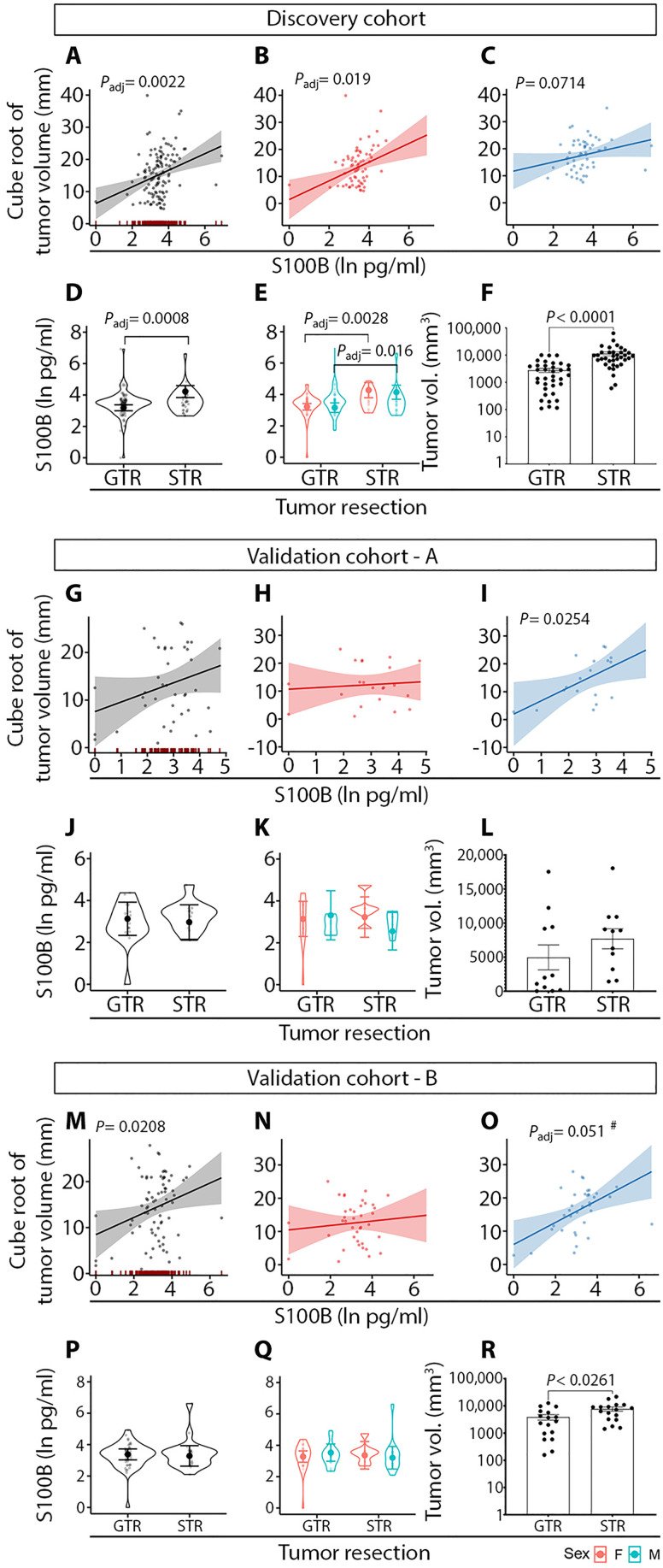
S100B is associated with preoperative tumor volume and is elevated in VS patients who underwent subtotal tumor resection. The discovery cohort (**A** to **F**) and validation cohort-A (**G** to **L**) were composed of 122 and 50 VS patients, respectively. Validation cohort-B (**M** to **R**) was created by augmenting validation cohort-A with 36 randomly selected patients out of 122 VS patients from the discovery cohort to reach a balanced number of patients in both groups (86 patients per group). GTR, gross tumor resection; STR, subtotal tumor resection; #, statistically significant at the FDR of 0.10. Gray, patients of both sexes; red, female sex; blue, male sex.

In the RLM assessing the relationship of biomarker levels and tumor volume, a significant association was found for S100B (Fig. 4A), with a significant sex effect among females (all *P*_adj_ < 0.05) (Fig. 4B). Furthermore, S100B levels were significantly elevated in VS patients who underwent subtotal resection (STR) rather than gross total resection (GTR) of VS (Fig. 4, D and E). In addition, tumor volume was higher in VS patients who underwent STR compared to GTR (Fig. 4F). In the validation cohort-A, there was a trend for a positive association between S100B plasma levels and tumor volume in male VS patients (Fig. 4I), which approached the criterion for statistical significance after correction for multiple comparisons by controlling the false discovery rate (FDR) to 0.10 (*P* = 0.025, critical value: 0.023 in male VS patients). In the balanced validation cohort-B (increased to *n* = 86 per the discovery cohort size), there was a significant association between S100B levels and tumor volume in male VS patients (*P*_adj_ = 0.051 was considered significant for FDR of 0.10) (Fig. 4O). However, the difference in S100B levels between the GTR and STR groups observed in the discovery cohort was not confirmed in the validation cohort, likely due to a smaller number of patients undergoing any VS surgical resection in the validation group (GTR, *n* = 17; STR, *n* = 11) (Fig. 4, J to L and P to R).

### Discriminative power, diagnostic utility, and relationships of candidate biomarkers

**Fig. 5.**
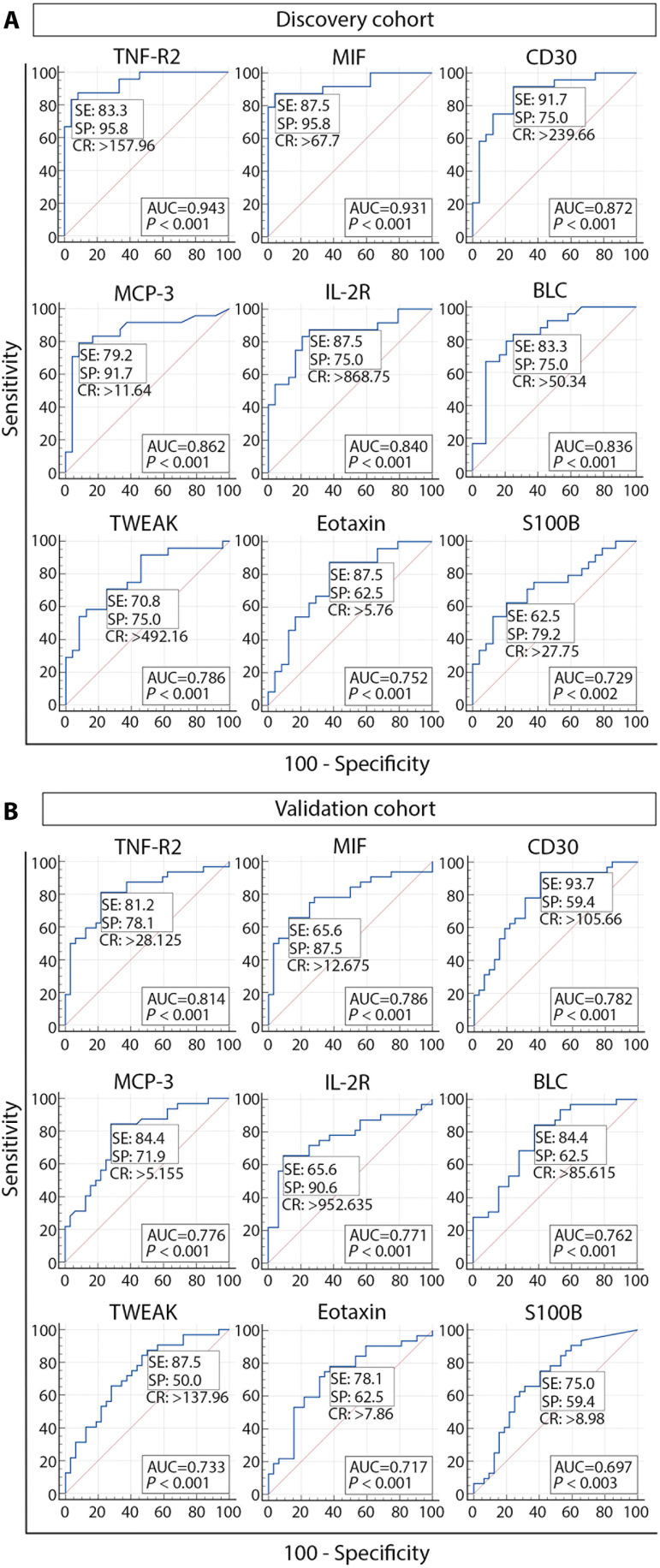
Diagnostic utility and discriminatory power of candidate biomarkers with significant AUC values. ROC analysis was performed in the discovery (**A**) and validation cohorts (**B**). AUC, area under the curve; SE, sensitivity; SP, specificity; CR, criterion.

In the receiver-operating characteristic curve (ROC) analysis of the balanced dataset of sex- and age-matched VS patients and controls, in both the discovery and validation cohorts, 9 of the 13 significantly elevated factors in VS patients’ plasma had a significant area under the curve (AUC) values. In the discovery cohort, AUC values were outstanding (0.9 to 1) for TNF-R2 and MIF; excellent (0.8 to 0.9) for CD30, MCP-3, IL-2R, and BLC; and acceptable (0.7 to 0.8) for TWEAK, eotaxin, and S100B. Although lower AUC values were observed in the validation cohort, the predictor capacity of tested biomarkers was still preserved, ranging from excellent for TNF-R2 to poor for S100B. In the validation cohort, TWEAK and eotaxin retained the unchanged predictor category, suggesting their predictor stability (Fig. 5). Moreover, the ranking of biomarkers in both cohorts remained unchanged, with TNF-R2 as a top biomarker and S100B with the lowest but statistically significant AUCs (0.729 and 0.697 in the discovery and validation cohorts, respectively). Consistent with our previously published data (*9*), the ROC analysis revealed that tumor size is not a robust biomarker of the hearing status in VS patients when comparing GH and PH (AUC = 0.543), or SH and NSH (AUC = 0.621).

To evaluate whether a biomarker panel could discriminate between VS patients and controls, a combinatorial analysis was performed using all nine validated biomarkers. The analysis revealed that combining nine biomarkers generated 502 panels ranging from two to nine biomarkers. Considering that MCP-3 and S100B are the only biomarkers significantly associated with patients’ preoperative hearing and tumor size, 128 panels containing both MCP-3 and S100B were analyzed. The distribution of biomarkers was monitored in the top 15 panels given that they have the best performance (discovery cohort: AUC higher than 0.991, with 95.8 to 100% sensitivity and specificity; validation cohort: AUC higher than 0.872, with 78.1 to 93.8% sensitivity and 65.6 to 84.4% specificity). The most abundant biomarkers were TNF-R2 (discovery cohort) and IL-2R (both cohorts). They were followed by BLC, eotaxin, TWEAK, MIF, and CD30 in the discovery cohort, while IL-2R was followed by eotaxin, TWEAK, TNF-R2, BLC, and MIF in the validation cohort. In both cohorts, the nine-biomarker panel is among the top 15 panels. It shares the first place with 11 other panels in the discovery cohort (AUC = 1, with 100% sensitivity and specificity) (table S3) and takes the sixth position in the validation cohort (AUC = 0.874, with 90.6% sensitivity and 75% specificity) (table S4).

**Fig. 6.**
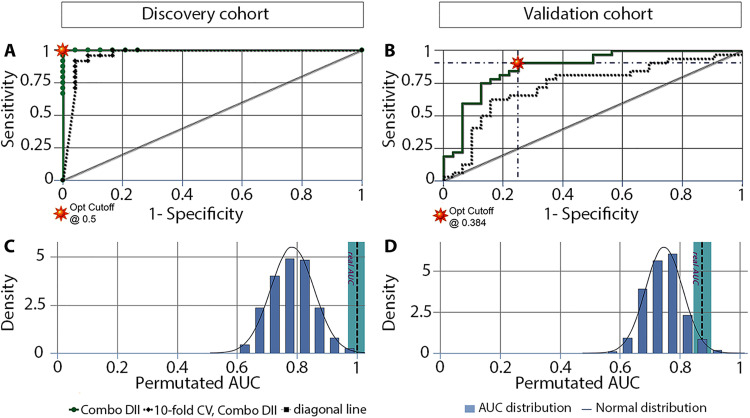
Tenfold cross-validation and permutation tests of the panel of nine candidate biomarkers selected as the best predictors associated with hearing and tumor size in VS patients. Tenfold cross-validation and permutation tests were performed in the discovery (**A** and **C**, respectively) and validation cohorts (**B** and **D**, respectively). The nine biomarkers in the panel (DII) were TNR-R2, MIF, CD30, MCP-3, IL-2R, BLC, TWEAK, eotaxin, and S100B. DII, biomarker combination 502 of 502; CV, cross-validation; Opt, optimal.

The nine-biomarker panel demonstrated significantly improved predictability compared to the mean AUC of the individual biomarkers (discovery cohort: AUC_9-panel_ = 1.000 ± 0.000 versus AUC_mean of 9_: 0.839 ± 0.025, *P* < 0.0001; validation cohort: AUC_9-panel_ = 0.890 ± 0.042 versus AUC_mean of 9_: 0.760 ± 0.012, *P* = 0.0029). This finding was confirmed by logistic regression analysis (table S5), and the accuracy and utility of the nine-biomarker panel were verified by performing 10-fold cross-validation and permutation tests (Fig. 6).

A mutual comparison of the nine candidate biomarkers with Spearman correlation analysis resulted in seven confirmed correlation pairs. Both VS patients and controls had significant positive correlations between (i) MCP-3 and BLC, (ii) CD30 and IL-2R, (iii) CD30 and MIF, and (iv) CD30 and TNF-R2. However, correlations between (i) BLC and TWEAK and (ii) CD30 and TWEAK were found mainly in VS patients, while the correlation between TNF-R2 and IL-2R was significant only in VS patients and not in controls, suggesting a disease-related association between these candidate biomarkers (fig. S9). The strongest positive correlations in VS patients were between MCP-3 and BLC (discovery cohort, *r =* 0.67; validation cohort, *r =* 0.71) and BLC and TWEAK (discovery cohort, *r =* 0.66; validation cohort, *r =* 0.82) (all *P* < 0.05). In healthy controls, the strongest positive correlation was observed between TNF-R2 and MIF (discovery cohort, *r =* 0.61; validation cohort, *r =* 0.87) (fig. S9).

## DISCUSSION

In this largest immune profiling to date of plasma from patients with sporadic VS, we identified 14 significantly elevated factors, 13 or which were verified in a separate cohort of VS patients. TNF-R2 was most highly elevated in VS patients, MCP-3 and S100B were associated with preoperative clinical characteristics, and nine factors showed a promising potential as circulating biomarkers.

Expression of TNF-R2 has been reported in many types of tumors, and serum concentration of soluble TNF-R2 is considered to be an indicator of TNF-R2 activity (*27*). Although TNF-R2 can be expressed on tumor cells, its main source may be highly suppressive regulatory T cells (T_regs_) because the expansion of T_regs_ in cancer patients is followed by elevated serum values of soluble TNF-R2 (*27*), consistent with immune evasion. Given that we did not detect an association between TNF-R2 plasma levels and tumor size or hearing status in VS patients, elevated TNF-R2 may reflect an increased number of immunosuppressive cells in the VS patients’ blood and more systemic immunosuppression than captured by tumor size or tumor-induced hearing loss. A significantly increased number of myeloid-derived suppressor cells have been reported in the blood of VS patients with *NF2*-related schwannomatosis (*28*). Future studies should determine whether there is a correlation between plasma levels of TNF-R2 and the number of circulatory T_regs_ in VS patients. In the meantime, our data support the notion of systemic immunosuppression in patients with sporadic VS because we found a significant positive correlation between TNF-R2 and IL-2R (*r* = 0.591, *P* = 0.002), known to be abundantly expressed on T_regs_ (*29*). Relevantly, intracranial malignancies have been previously associated with systemic immunosuppression (*30*), which in turn has been associated with hearing impairment (*31*).

MCP-3 is an important immune-related factor whose relationship with GH is highlighted by our study. We showed that, in VS patients, MCP-3 is significantly elevated among those with SH and that higher MCP-3 levels were associated with better WR scores. It is currently unknown whether MCP-3, a proinflammatory chemokine with a prominent role in tumorigenesis (*32*), could be involved in hearing protection. Considering that this chemokine performs its function by binding to four receptors (CCR1, CCR2, CCR3, and CCR5), which are shared with other chemokines (*33*), MCP-3 may antagonize other chemokines with potential ototoxic activity. It is interesting that a receptor for MCP-3 is CCR2, which is reported to play a protective role against noise-induced cochlear hair cell death (*34*). Moreover, DARC, an atypical chemokine receptor that can bind MCP-3, is broadly expressed in the mouse cochlea, including hair cells, supporting cells, and spiral ganglion neurons (*35*). This finding suggests that tumor-secreted MCP-3 may affect cochlear function.

S100B is a candidate biomarker whose plasma values were significantly associated with tumor volume. S100B is broadly expressed in Schwann cells whose proliferation, myelinating activity, and S100B expression are controlled by the *SOX-10* transcription factor (*36*). Reduced expression of *SOX-10* at the protein and gene levels has been demonstrated in human schwannoma cells, while the loss of *SOX-10* expression in Schwann cells leads to them acquiring schwannoma cell characteristics (*37*). While the direct effect of S100B on schwannoma growth has not been studied mechanistically, it has been established that the protumorigenic effect of S100B in melanoma and glioma involves inhibition of p53 tumor suppressor protein activity, stimulation of mitogenic kinases activity (Ndr and Akt), and macrophage chemoattraction (*38*, *39*). A correlation between S100B serum levels and tumor growth has been reported in melanoma patients, where decreased levels were associated with clinical response to therapy (*40*). For VS, there are two reports showing opposite outcomes. Similar to us, Kanner *et al.* (*41*) reported a significant positive correlation between serum S100B levels and tumor size only for VS tumors (*n* = 6) but not for all other analyzed solid tumors, including glial tumors (*n* = 8), metastatic tumors (*n* = 27, predominantly lung and breast carcinoma), meningiomas (*n* = 8), and chondroma (*n* = 1). However, Smith *et al.* (*14*) reported that plasma S100B was not a useful biomarker for tumor burden in neurofibromatosis patients (*NF1*-related schwannomatosis: *n* = 69; *NF2*-related schwannomatosis: *n* = 28; schwannomatosis: *n* = 30) because they did not find a relationship between the presence of internal neurofibromas or schwannomas and S100B plasma levels, or between whole-body tumor burden and S100B concentration. However, Smith *et al.* noted the limited ability of whole-body MRI to image the internal auditory canal, which prevented them from confirming any relationship between VS size and S100B levels in patients with *NF2*-related schwannomatosis (*14*). Together, our plasma findings measured in 122 patents with sporadic VS, along with the results of Kanner *et al.*, suggest the utility of S100B plasma levels as a biomarker associated with tumor size in VS patients.

Finally, we demonstrated and validated the diagnostic utility of 9 of 13 immune-related factors with confirmed significantly elevated levels in VS patients. We proposed a nine-biomarker panel and showed its excellent discrimination ability for VS. TNF-R2 was chosen as the highly elevated biomarker in VS patients, MCP-3 was selected as the only verified factor significantly associated with SH and WR, S100B is a phenotypic marker of VS correlating with tumor size, and MIF, CD30, IL-2R, BLC, TWEAK, and eotaxin were selected based on their contribution to improving the predictability (panel AUC) of the other biomarkers. Correlation analysis showed significant interconnections between the panel biomarkers, suggesting possible roles in VS pathogenesis. Overall, our findings suggest that the nine-biomarker panel could be an additional tool to monitor or predict hearing change and tumor growth in VS patients and help inform diagnoses or the ideal timing of tumor resection to preserve hearing.

One limitation of our study is that it is cross-sectional, and therefore, the temporal link between the outcome and VS presence cannot be determined because both are examined simultaneously. A future longitudinal study focused on the validation of our findings is suggested. Such a study may benefit from multi-institutional patient accrual because VS is a rare tumor. Another limitation of our study is the lack of quantitative hearing assessment in control patients because their deidentified blood was collected from blood donor centers. However, we quantified hearing in ears contralateral to VS (fig. S1), and hearing in these non–tumor-bearing ears of VS-GH patients should be representative of the control population that is similar in age and sex distributions, as we previously showed (*21*). In that study, we also showed that VS-PH patients have long-term risk of progression to moderate hearing loss in the contralateral ear as well, which typically manifests 12 years after VS-PH diagnosis (*21*). We validated differences in candidate biomarkers between VS patients and controls in two different patient populations and two different laboratories. Nonetheless, a future study involving quantitative audiometric data for all control subjects is suggested to discern how hearing status may influence immune-related molecules in plasma.

In summary, this study describes the robust immune profiling of blood plasma from a large cohort of patients with sporadic VS for comparison with controls, between VS-GH and VS-PH patients, between VS patients with SH and NSH, and between sexes. We correlated biomarker levels with presurgical PTA, WR, and tumor size and validated a potentially diagnostic nine-biomarker composite panel with outstanding/excellent discriminatory ability for VS.

## MATERIALS AND METHODS

### Study population and specimen collection

From July 2015 to April 2021, blood was prospectively collected from patients undergoing VS resection at Massachusetts Eye and Ear (MEE) in Boston, MA on the day of surgery, typically within 30 min of inducing general anesthesia and ≥1 hour before tumor microdissection (discovery cohort). Blood was similarly prospectively collected from August 2021 to June 2023 at Stanford Hospital in Palo Alto, CA (validation cohort). Blood from controls was collected at the Massachusetts General Hospital (MGH) Blood Donor Center in Boston, MA, and the Research Blood Components in Watertown, MA for the discovery cohort, and from the Stanford Hospital Blood Donor Center in Palo Alto, CA for the validation cohort. At collection, fresh blood was stored in EDTA vacutainer tubes (Becton Dickinson, NY, USA) and kept at 4°C without freezing. The whole blood samples were centrifuged at 2000*g* for 10 min at 4°C. Plasma was separated and spun at 2000*g* for 5 min at 4°C. Centrifuged plasma was filtered through 0.8-μm filter units (MF-Millipore MCE membrane, SLAA033SB; Millipore, Burlington, MA, USA) and stored at −80°C until further use.

Eligible patients had unilateral, sporadic VS that had not been previously resected or irradiated. Of 186 and 50 enrolled VS patients in the discovery and validation cohorts, 163 and 50 met inclusion criteria, respectively, and were included in the analyses (CONSORT diagram in fig. S1) for comparison with 70 and 43 controls, respectively.

### Clinical data

Clinical and demographic data were collected from patient charts, operative reports, pathology reports, and preoperative radiographic imaging. Patient variables included age at tissue collection, presurgical tumor volume measured via high-resolution axial contrast-enhanced T1-weighted brain MRI, internal auditory canal protocol (*42*), and presurgical pure-tone audiometric threshold and WR measurements. MRI and hearing tests were those nearest to resection, typically ≤3 months. WR was defined as the percentage of spoken monosyllabic words discernable from a list typically read at 70 dB or the level at which a patient’s speech intelligibility curve plateaus. Pure-tone audiometric thresholds at 0.5, 1, 2, and 3 kHz were used to calculate the PTA. Hearing groups were defined according to the American Academy of Otolaryngology–Head and Neck Surgery (AAO-HNS) Hearing Classification Guidelines (*43*). GH was defined as WR > 70% and PTA < 30 dB (AAO-HNS class A hearing). Otherwise, patients were classified as having PH (AAO-HNS class B, C, and D hearing). A deaf ear was assigned a PTA of 125 dB and WR score of 0%. SH was defined as WR ≥ 50% and PTA ≤ 50 dB (AAO-HNS class A and B hearing). Otherwise, patients were classified as having NSH (AAO-HNS class C and D hearing). Tumor resection was defined as GTR if there was complete tumor removal or a small tumor remnant no greater than 5 × 5 × 2 mm was left behind to preserve nerve integrity (*17*). Otherwise, it was defined as STR.

### Biomarker measurements

Luminex (65 cytokines, chemokines, growth factors, and soluble receptors), electrochemiluminescence (IL-18, TNF-α, and FGF-2), and ELISA (enzyme-linked immunosorbent assay) (S100B) assays were conducted as described in detail below. Potential biomarkers were defined as those detectable in the plasma of >75% of patients. The list of analyzed biomarkers is in table S1.

### Luminex assay

Simultaneous multiplex profiling of 65 immune-related factors composed of cytokines, chemokines, and growth factors was performed using a customized multiplex bead-based immunoassay—Immune Monitoring 65-Plex Human ProcartaPlex Panel (#EPX650-10065-901; Thermo Fisher Scientific, Waltham, MA, USA) according to the manufacturer’s instructions. The fluorescence-based signal was acquired on the Magpix instrument (Luminex, Austin, TX, USA), and the values of analytes were calculated using ProcartaPlex Analyst 1.0 Software (Thermo Fisher Scientific). The analytes with values below the lower limit of quantification in more than 75% of all samples were excluded from the analysis (table S1). For the accepted analytes, the values between 0 pg/ml and the lower limit of quantification, and those exceeding the upper limit of quantification, were approximated with the lowest and highest concentration representing these limits, respectively.

### Electrochemiluminescence assay

The following candidate biomarkers were measured using electrochemiluminescence-based human assays from Meso Scale Diagnostics (MSD; Rockville, MD, USA): IL-18 (U-PLEX Human IL-18) and TNF-α (U-PLEX Human TNF-α). All assays were conducted according to the manufacturer’s protocols, and signal detection was performed on the QuickPlex SQ 120 device (MSD). Preanalytical data processing was done using MSD Discovery Workbench software (v4.0.12).

### Enzyme-linked immunosorbent assay

The S100B ELISA Kit (#EZHS100B- 33K; EMD Millipore, Billerica, MA, USA) was used to measure S100B protein levels in the plasma of VS patients and controls. ELISAs were performed by adhering to the manufacturer’s protocol. Absorbance was measured using SpectraMax 190 plate Reader (Molecular Devices, Sunnyvale, CA, USA), and the standard curve was plotted using SoftMax Pro software (v5.2; Molecular Devices, San Jose, CA, USA).

### Statistics

Patient group comparisons comprised (i) all VS patients versus controls), (ii) VS-GH patients versus controls, (iii) VS-PH patients versus controls, (iv) VS-PH versus VS-GH patients, (v) VS-SH versus VS-NSH, and (vi) VS patients undergoing GTR versus STR. For all comparisons, *P* < 0.05 was considered statistically significant. *P* values were corrected for multiple comparisons (and called *P*_adj_) using the Benjamini-Hochberg FDR procedure. Differences in age across groups were analyzed using one-way analysis of variance (ANOVA) with a Dunn’s multiple comparisons test. Differences in tumor volume and PTA were analyzed using Mann-Whitney *t* test. Differences in WR were analyzed using *N* − 1 chi-square test for the comparison of two proportions expressed as a percentage. The ratio method was used to assess the individual effect of candidate biomarkers on the WR of VS patients.

### Generalized linear mixed-effects regression

Generalized linear mixed-effects regression models were used to assess the relationship between candidate biomarkers’ levels in the plasma of VS patients and controls versus clinical variables. All candidate biomarkers were natural log-transformed before modeling. To compare plasma biomarker levels in VS patients with controls while controlling for subjects’ age and sex, the following generalized least squares (GLS) model was used: *ln<biomarker>* = β0 + β1*Sex* + β2*Age* + β*GH* + β4*PH*. The biomarker in the formula refers to tested immune-related factors, while β0, β1, β2, β3, and β4 are regression coefficients representing the change in natural log-transformed concentrations of prospective biomarkers to a one-unit change in the respective independent variable (sex, age, GH, and PH). The effect of sex × candidate biomarker interactions was assessed by the same model with the following terms: *ln<biomarker> *= β0 + β1*Sex* + β2*Age* + β3*GH* + β4*PH* + β5*Sex* × *ln<biomarker>*. For mutual comparison of GH and PH groups, the GLS model was extended for controlling the subject’s tumor volume.

RLMs were used to regress ipsilateral PTA values on plasma biomarker levels while controlling for sex, age, tumor volume, and PTA contralateral to VS. RLMs were also used to regress tumor volume on plasma biomarker levels while controlling for sex and age. The use of RLMs was necessary for both outcomes, as non-RLMs suffered from substantial effect size biases caused by residual outliers. RLM was used to control for patients’ age, sex, tumor size, and hearing in the contralateral ear. The model had the following terms: *PTA_ipsi* = β0 + β1*Sex* + β2*Age* + β3*PTA_contra* + β4*Tumor volume*^(1/3) + β5*ln<biomarker>*, where *PTA_ipsi* is PTA in the ear ipsilateral to the tumor and *PTA_contra* is PTA in the ear contralateral to the tumor. To investigate the effect of sex × candidate biomarker interactions, the same RLM was used with the following terms: *PTA_ipsi* = β0 + β1*Sex* + β2*Age* + β3*PTA_contra* + β4*Tumor volume*^(1/3) + β5*ln<biomarker>* + β6*Sex* × *ln<biomarker>*. The regression coefficients (β0, β1, β2, β3, β4, β5, and β6) represent the change in *PTA_ipsi *to a one-unit change in the respective independent variable (sex, age, *PTA_contra*, tumor volume, biomarker, and interactions of sex and biomarker concentration).

In the analysis of the interaction with tumor volume, the RLM had the following terms. *Tumor volume*^(1/3) = β0 + β1*Sex* + β2*Age* + β3*ln<biomarker>*, where β0, β1, β2, and β3 are regression coefficients representing the change in tumor volume to a one-unit change in the respective independent variable (sex, age, and biomarker concentration). To investigate the effect of sex × candidate biomarker interactions, the same RLM was extended as follows: *Tumor volume*^(1/3) = β0 + β1*Sex* + β2*Age* + β3*ln<biomarker>* + β4*Sex* × *<ln biomarker>*, where β4 is a regression coefficient representing the change in tumor volume to a one-unit change in the variable determined by interactions of sex and biomarker concentration.

FLMs were used to regress WR scores ipsilateral to VS on plasma biomarker levels while controlling for sex, age, tumor volume, and WR scores contralateral to VS. Fractional models were necessary since WR scores were measured on the interval [0, 100] and therefore had hard upper and lower bounds, as well as being heteroskedastic. FLM was used to control for patients’ age, sex, tumor size, and hearing in the contralateral ear. The model had the following terms: *WR_ipsi* = β0 + β1*Sex* + β2*Age* + β3*WR_contra* + β4*Tumor volume*^(1/3) + β5*ln<biomarker>*, where *WR_ipsi* is WR in the ear ipsilateral to the tumor and *WR_contra* is WR in the ear contralateral to the tumor. The effect of sex × candidate biomarker interactions was assessed by the same model with the following terms: *WR_ipsi* = β0 + β1*Sex* + β2*Age* + β3*WR_contra* + β4*Tumor volume*^(1/3) + β5*ln<biomarker>* + β6*Sex* × *ln<biomarker>*. The regression coefficients (β0, β1, β2, β3, β4, β5, and β6) represent the change in *WR_ipsi* to a one-unit change in the respective independent variable (sex, age, *WR_contra*, tumor volume, biomarker, and interactions of sex and biomarker concentration).

ROC analysis was used to evaluate the diagnostic power and utility of significantly elevated candidate plasma biomarkers. The ROC analysis was performed on the balanced dataset of sex- and age-matched VS patients and controls, and the areas under the ROC curve (AUCs) were calculated using MedCalc software. The discriminatory power of candidate biomarkers was categorized as follows: “outstanding” discrimination, AUC ≥ 0.90; “excellent” discrimination, 0.80 ≤ AUC < 0.90; “acceptable” discrimination, 0.70 ≤ AUC < 0.80; and “poor” discrimination, AUC < 0.70 (*44*).

### Statistical software

Comparisons of demographics, tumor volume, and hearing loss between groups were performed with GraphPad Prism (v9.3.1; GraphPad Software, La Jolla, CA, USA) and MedCalc Statistical Software (v20.109; MedCalc Software Ltd., Ostend, Belgium). Models were estimated, and graphs were generated using R (v3.6.3; R Foundation, Vienna, Austria). Combinatorial analysis of candidate biomarkers was performed using the CombiROC web application (http://combiroc.eu) (*45*) and verified by logistic regression with the enter method using MedCalc software. The relationship between panel biomarkers was assessed by Spearman correlation analysis using GraphPad software. 

### Study approval

All study protocols were approved by the Human Studies Committee of MEEI and MGH [Institutional Review Board (IRB) protocol #14-148H] as well as Stanford (IRB protocol #60363). All participants provided written informed consent before participation.

## References

[1] M. S. Mahaley Jr., C. Mettlin, N. Natarajan, E. R. Laws Jr., B. B. Peace, Analysis of patterns of care of brain tumor patients in the United States: A study of the Brain Tumor Section of the AANS and the CNS and the Commission on Cancer of the ACS. Clin. Neurosurg. 36, 347–352 (1990). 2295209

[2] R. A. Sobel, Vestibular (acoustic) schwannomas: Histologic features in neurofibromatosis 2 and in unilateral cases. J. Neuropathol. Exp. Neurol. 52, 106–113 (1993). 8440992 10.1097/00005072-199303000-00002

[3] J. L. Fisher, D. Pettersson, S. Palmisano, J. A. Schwartzbaum, C. G. Edwards, T. Mathiesen, M. Prochazka, T. Bergenheim, R. Florentzson, H. Harder, G. Nyberg, P. Siesjö, M. Feychting, Loud noise exposure and acoustic neuroma. Am. J. Epidemiol. 180, 58–67 (2014). 24786799 10.1093/aje/kwu081

[4] S. R. Plotkin, L. Messiaen, E. Legius, P. Pancza, R. A. Avery, J. O. Blakeley, D. Babovic-Vuksanovic, R. Ferner, M. J. Fisher, J. M. Friedman, M. Giovannini, D. H. Gutmann, C. O. Hanemann, M. Kalamarides, H. Kehrer-Sawatzki, B. R. Korf, V. F. Mautner, M. MacCollin, L. Papi, K. A. Rauen, V. Riccardi, E. Schorry, M. J. Smith, A. Stemmer-Rachamimov, D. A. Stevenson, N. J. Ullrich, D. Viskochil, K. Wimmer, K. Yohay; International Consensus Group on Neurofibromatosis Diagnostic Criteria (I-NF-DC), S. M. Huson, P. Wolkenstein, D. G. Evans, Updated diagnostic criteria and nomenclature for neurofibromatosis type 2 and schwannomatosis: An international consensus recommendation. Genet. Med. 24, 1967–1977 (2022). 35674741 10.1016/j.gim.2022.05.007

[5] C. Matthies, M. Samii, Management of 1000 vestibular schwannomas (acoustic neuromas): Clinical presentation. Neurosurgery 40, 1–9 (1997). 8971818 10.1097/00006123-199701000-00001

[6] D. J. Genther, K. D. Frick, D. Chen, J. Betz, F. R. Lin, Association of hearing loss with hospitalization and burden of disease in older adults. JAMA 309, 2322–2324 (2013). 23757078 10.1001/jama.2013.5912 PMC3875309

[7] A. Mohammadi, N. Jufas, Sudden death due to vestibular schwannoma: Caution in emergent management. Otol. Neurotol. 37, 564–567 (2016). 26945316 10.1097/MAO.0000000000001004

[8] E. E. Smouha, M. Yoo, K. Mohr, R. P. Davis, Conservative management of acoustic neuroma: A meta-analysis and proposed treatment algorithm. Laryngoscope 115, 450–454 (2005). 15744156 10.1097/00005537-200503000-00011

[9] A. Brown, S. Early, S. Vasilijic, K. M. Stankovic, Sporadic vestibular schwannoma size and location do not correlate with the severity of hearing loss at initial presentation. Front. Oncol. 12, 836504 (2022). 35372070 10.3389/fonc.2022.836504 PMC8965062

[10] B. Akpinar, S. H. Mousavi, M. M. McDowell, A. Niranjan, A. H. Faraji, J. C. Flickinger, L. D. Lunsford, Early radiosurgery improves hearing preservation in vestibular schwannoma patients with normal hearing at the time of diagnosis. Int. J. Radiat. Oncol. Biol. Phys. 95, 729–734 (2016). 26975929 10.1016/j.ijrobp.2016.01.019

[11] M. E. Sughrue, A. J. Kane, R. Kaur, J. J. Barry, M. J. Rutkowski, L. H. Pitts, S. W. Cheung, A. T. Parsa, A prospective study of hearing preservation in untreated vestibular schwannomas. J. Neurosurg. 114, 381–385 (2011). 20486891 10.3171/2010.4.JNS091962

[12] M. E. Sughrue, I. Yang, D. Aranda, K. Lobo, L. H. Pitts, S. W. Cheung, A. T. Parsa, The natural history of untreated sporadic vestibular schwannomas: A comprehensive review of hearing outcomes. J. Neurosurg. 112, 163–167 (2010). 19538047 10.3171/2009.4.JNS08895

[13] S. R. Plotkin, J. O. Blakeley, E. Dombi, M. J. Fisher, C. O. Hanemann, K. S. Walsh, P. L. Wolters, B. C. Widemann, Achieving consensus for clinical trials: The REiNS International Collaboration. Neurology 81 (21 Suppl. 1), S1–S5 (2013). 24249801 10.1212/01.wnl.0000435743.49414.b6 PMC3908338

[14] M. J. Smith, S. Esparza, V. L. Merker, A. Muzikansky, M. A. Bredella, G. J. Harris, A. Kassarjian, W. Cai, J. A. Walker, V. F. Mautner, S. R. Plotkin, Plasma S100β is not a useful biomarker for tumor burden in neurofibromatosis. Clin. Biochem. 46, 698–700 (2013). 23261835 10.1016/j.clinbiochem.2012.12.007

[15] J. O. Blakeley, X. Ye, D. G. Duda, C. F. Halpin, A. L. Bergner, A. Muzikansky, V. L. Merker, E. R. Gerstner, L. M. Fayad, S. Ahlawat, M. A. Jacobs, R. K. Jain, C. Zalewski, E. Dombi, B. C. Widemann, S. R. Plotkin, Efficacy and biomarker study of bevacizumab for hearing loss resulting from neurofibromatosis type 2-associated vestibular schwannomas. J. Clin. Oncol. 34, 1669–1675 (2016). 26976425 10.1200/JCO.2015.64.3817 PMC4872317

[16] S. R. Plotkin, D. G. Duda, A. Muzikansky, J. Allen, J. Blakeley, T. Rosser, J. L. Campian, D. W. Clapp, M. J. Fisher, J. Tonsgard, N. Ullrich, C. Thomas, G. Cutter, B. Korf, R. Packer, M. A. Karajannis, Multicenter, prospective, phase II and biomarker study of high-dose bevacizumab as induction therapy in patients with neurofibromatosis type 2 and progressive vestibular schwannoma. J. Clin. Oncol. 37, 3446–3454 (2019). 31626572 10.1200/JCO.19.01367 PMC7098833

[17] Y. Ren, H. Hyakusoku, J. E. Sagers, L. D. Landegger, D. B. Welling, K. M. Stankovic, MMP-14 (MT1-MMP) is a biomarker of surgical outcome and a potential mediator of hearing loss in patients with vestibular schwannomas. Front. Cell. Neurosci. 14, 191 (2020). 32848608 10.3389/fncel.2020.00191 PMC7424165

[18] S. Dilwali, A. Lysaght, D. Roberts, F. G. Barker II, M. J. McKenna, K. M. Stankovic, Sporadic vestibular schwannomas associated with good hearing secrete higher levels of fibroblast growth factor 2 than those associated with poor hearing irrespective of tumor size. Otol. Neurotol. 34, 748–754 (2013). 23512073 10.1097/MAO.0b013e31828048ec PMC3655133

[19] S. Dilwali, L. D. Landegger, V. Y. Soares, D. G. Deschler, K. M. Stankovic, Secreted factors from human vestibular schwannomas can cause cochlear damage. Sci. Rep. 5, 18599 (2015). 26690506 10.1038/srep18599 PMC4686978

[20] J. E. Sagers, M. I. Sahin, I. Moon, S. G. Ahmed, A. Stemmer-Rachamimov, G. J. Brenner, K. M. Stankovic, NLRP3 inflammasome activation in human vestibular schwannoma: Implications for tumor-induced hearing loss. Hear. Res. 381, 107770 (2019). 31430634 10.1016/j.heares.2019.07.007

[21] S. Early, C. E. Rinnooy Kan, M. Eggink, J. H. M. Frijns, K. M. Stankovic, Progression of contralateral hearing loss in patients with sporadic vestibular schwannoma. Front. Neurol. 11, 796 (2020). 33013614 10.3389/fneur.2020.00796 PMC7461819

[22] L. Koelman, O. Pivovarova-Ramich, A. F. H. Pfeiffer, T. Grune, K. Aleksandrova, Cytokines for evaluation of chronic inflammatory status in ageing research: Reliability and phenotypic characterisation. Immun. Ageing 16, 11 (2019). 31139232 10.1186/s12979-019-0151-1 PMC6530020

[23] S. E. O'Bryant, S. Lista, R. A. Rissman, M. Edwards, F. Zhang, J. Hall, H. Zetterberg, S. Lovestone, V. Gupta, N. Graff-Radford, R. Martins, A. Jeromin, S. Waring, E. Oh, M. Kling, L. D. Baker, H. Hampel, Comparing biological markers of Alzheimer's disease across blood fraction and platforms: Comparing apples to oranges. Alzheimer's Dement. 3, 27–34 (2016). 10.1016/j.dadm.2015.12.003 PMC4802360 27019866

[24] W. Hong, M. Zhao, H. Li, F. Peng, F. Wang, N. Li, H. Xiang, Y. Su, Y. Huang, S. Zhang, G. Zhao, R. Zhou, L. Mao, Z. Lin, Y. Fang, Q. Zhang, B. Xie, Higher plasma S100B concentrations in schizophrenia patients, and dependently associated with inflammatory markers. Sci. Rep. 6, 27584 (2016). 27279465 10.1038/srep27584 PMC4899785

[25] S. Sugie, S. Mukai, K. Yamasaki, T. Kamibeppu, H. Tsukino, T. Kamoto, Plasma macrophage-stimulating protein and hepatocyte growth factor levels are associated with prostate cancer progression. Hum. Cell 29, 22–29 (2016). 26250899 10.1007/s13577-015-0123-5

[26] M. Beranek, P. Kolar, S. Tschoplova, K. Kankova, A. Vasku, Genetic variation and plasma level of the basic fibroblast growth factor in proliferative diabetic retinopathy. Diabetes Res. Clin. Pract. 79, 362–367 (2008). 17997184 10.1016/j.diabres.2007.09.012

[27] É. S. Vanamee, D. L. Faustman, TNFR2: A novel target for cancer immunotherapy. Trends Mol. Med. 23, 1037–1046 (2017). 29032004 10.1016/j.molmed.2017.09.007

[28] Y. Wang, P. Li, B. Wang, S. Wang, P. Liu, Identification of myeloid-derived suppressor cells that have an immunosuppressive function in NF2 patients. J. Cancer Res. Clin. Oncol. 145, 523–533 (2019). 30603902 10.1007/s00432-018-02825-8 PMC11810391

[29] T. Chinen, A. K. Kannan, A. G. Levine, X. Fan, U. Klein, Y. Zheng, G. Gasteiger, Y. Feng, J. D. Fontenot, A. Y. Rudensky, An essential role for the IL-2 receptor in T_reg_ cell function. Nat. Immunol. 17, 1322–1333 (2016). 27595233 10.1038/ni.3540 PMC5071159

[30] P. Chongsathidkiet, C. Jackson, S. Koyama, F. Loebel, X. Cui, S. H. Farber, K. Woroniecka, A. A. Elsamadicy, C. A. Dechant, H. R. Kemeny, L. Sanchez-Perez, T. A. Cheema, N. C. Souders, J. E. Herndon, J.-V. Coumans, J. I. Everitt, B. V. Nahed, J. H. Sampson, M. D. Gunn, R. L. Martuza, G. Dranoff, W. T. Curry, P. E. Fecci, Sequestration of T cells in bone marrow in the setting of glioblastoma and other intracranial tumors. Nat. Med. 24, 1459–1468 (2018). 30104766 10.1038/s41591-018-0135-2 PMC6129206

[31] A. Zhang, T. Zou, D. Guo, Q. Wang, Y. Shen, H. Hu, B. Ye, M. Xiang, The immune system can hear noise. Front. Immunol. 11, 619189 (2020). 33679706 10.3389/fimmu.2020.619189 PMC7930229

[32] Y. Liu, Y. Cai, L. Liu, Y. Wu, X. Xiong, Crucial biological functions of CCL7 in cancer. PeerJ 6, e4928 (2018). 29915688 10.7717/peerj.4928 PMC6004300

[33] A. Zlotnik, O. Yoshie, The chemokine superfamily revisited. Immunity 36, 705–716 (2012). 22633458 10.1016/j.immuni.2012.05.008 PMC3396424

[34] N. B. Sautter, E. H. Shick, R. M. Ransohoff, I. F. Charo, K. Hirose, CC chemokine receptor 2 is protective against noise-induced hair cell death: Studies in CX3CR1^+/GFP^ mice. J. Assoc. Res. Otolaryngol. 7, 361–372 (2006). 17075702 10.1007/s10162-006-0051-x PMC2504633

[35] L. D. Landegger, S. Vasilijic, T. Fujita, V. Y. Soares, R. Seist, L. Xu, K. M. Stankovic, Cytokine levels in inner ear fluid of young and aged mice as molecular biomarkers of noise-induced hearing loss. Front. Neurol. 10, 977 (2019). 31632328 10.3389/fneur.2019.00977 PMC6749100

[36] S. Fujiwara, S. Hoshikawa, T. Ueno, M. Hirata, T. Saito, T. Ikeda, H. Kawaguchi, K. Nakamura, S. Tanaka, T. Ogata, SOX10 transactivates S100B to suppress Schwann cell proliferation and to promote myelination. PLOS ONE 9, e115400 (2014). 25536222 10.1371/journal.pone.0115400 PMC4275212

[37] R. D. S. Doddrell, X.-P. Dun, A. Shivane, M. L. Feltri, L. Wrabetz, M. Wegner, E. Sock, C. O. Hanemann, D. B. Parkinson, Loss of SOX10 function contributes to the phenotype of human Merlin-null schwannoma cells. Brain 136, 549–563 (2013). 23413263 10.1093/brain/aws353 PMC3572932

[38] J. Lin, Q. Yang, Z. Yan, J. Markowitz, P. T. Wilder, F. Carrier, D. J. Weber, Inhibiting S100B restores p53 levels in primary malignant melanoma cancer cells. J. Biol. Chem. 279, 34071–34077 (2004). 15178678 10.1074/jbc.M405419200

[39] H. Wang, L. Zhang, I. Y. Zhang, X. Chen, A. Da Fonseca, S. Wu, H. Ren, S. Badie, S. Sadeghi, M. Ouyang, C. D. Warden, B. Badie, S100B promotes glioma growth through chemoattraction of myeloid-derived macrophages. Clin. Cancer Res. 19, 3764–3775 (2013). 23719262 10.1158/1078-0432.CCR-12-3725 PMC3725731

[40] A. R. Bresnick, D. J. Weber, D. B. Zimmer, S100 proteins in cancer. Nat. Rev. Cancer 15, 96–109 (2015). 25614008 10.1038/nrc3893 PMC4369764

[41] A. A. Kanner, N. Marchi, V. Fazio, M. R. Mayberg, M. T. Koltz, V. Siomin, G. H. J. Stevens, T. Masaryk, B. Aumayr, M. A. Vogelbaum, G. H. Barnett, D. Janigro, Serum S100β: A noninvasive marker of blood-brain barrier function and brain lesions. Cancer 97, 2806–2813 (2003). 12767094 10.1002/cncr.11409 PMC4135471

[42] C. K. Kandathil, M. E. Cunnane, M. J. McKenna, H. D. Curtin, K. M. Stankovic, Correlation between aspirin intake and reduced growth of human vestibular schwannoma: Volumetric analysis. Otol. Neurotol. 37, 1428–1434 (2016). 27631829 10.1097/MAO.0000000000001180

[43] Committee on Hearing and Equilibrium, Committee on Hearing and Equilibrium guidelines for the evaluation of hearing preservation in acoustic neuroma (vestibular schwannoma). American Academy of Otolaryngology-Head and Neck Surgery Foundation, INC. Otolaryngol. Head Neck Surg. 113, 179–180 (1995). 7675475 10.1016/S0194-5998(95)70101-X

[44] D. W. Hosmer Jr., S. Lemeshow, R. X. Sturdivant, *Applied Logistic Regression* (John Wiley & Sons, ed. 3, 2013).

[45] S. Mazzara, R. L. Rossi, R. Grifantini, S. Donizetti, S. Abrignani, M. Bombaci, CombiROC: An interactive web tool for selecting accurate marker combinations of omics data. Sci. Rep. 7, 45477 (2017). 28358118 10.1038/srep45477 PMC5371980

