# Supplementary material for: Identification of immune-related candidate biomarkers in plasma of patients with sporadic vestibular schwannoma
Source: Sci Adv. 2023 Nov 10;9(45):eadf7295. doi: 10.1126/sciadv.adf7295 (PMC10637750; doi:10.1126/sciadv.adf7295)
Supplement: Supplementary file 1 — Figs. S1 to S7 Tables S1 to S5 [file sciadv.adf7295_sm.pdf]

Science Advances
AAAS

Supplementary Materials for

Identification of immune-related candidate biomarkers in plasma of patients with sporadic vestibular schwannoma

Sasa Vasilijic *et al.*

Corresponding author: Konstantina M. Stankovic, kstankovic@stanford.edu

Sci. Adv. **9**, eadf7295 (2023)
DOI: 10.1126/sciadv.adf7295

This PDF file includes:

Figs. S1 to S7
Tables S1 to S5

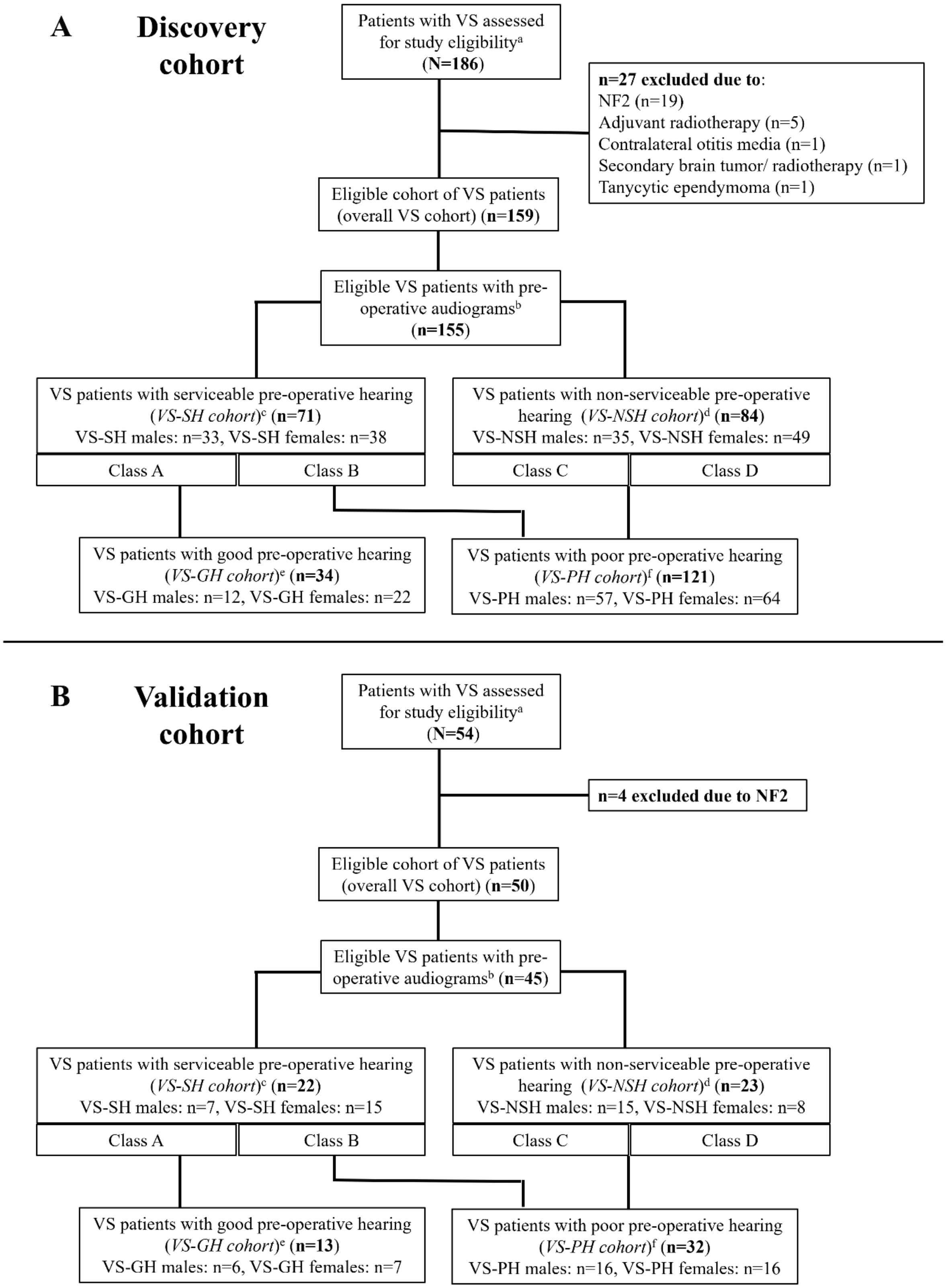
A
Discovery cohort
Patients with VS assessed for study eligibility[a] (N=186)
n=27 excluded due to:
NF2 (n=19)
Adjuvant radiotherapy (n=5)
Contralateral otitis media (n=1)
Secondary brain tumor/ radiotherapy (n=1)
Tanycytic ependymoma (n=1)
Eligible cohort of VS patients (overall VS cohort) (n=159)
Eligible VS patients with pre-operative audiograms[b] (n=155)
VS patients with serviceable pre-operative hearing (VS-SH cohort)[c] (n=71)
VS-SH males: n=33, VS-SH females: n=38
VS patients with non-serviceable pre-operative hearing (VS-NSH cohort)[d] (n=84)
VS-NSH males: n=35, VS-NSH females: n=49
Class A
Class B
Class C
Class D
VS patients with good pre-operative hearing (VS-GH cohort)[e] (n=34)
VS-GH males: n=12, VS-GH females: n=22
VS patients with poor pre-operative hearing (VS-PH cohort)[f] (n=121)
VS-PH males: n=57, VS-PH females: n=64
B
Validation cohort
Patients with VS assessed for study eligibility[a] (N=54)
n=4 excluded due to NF2
Eligible cohort of VS patients (overall VS cohort) (n=50)
Eligible VS patients with pre-operative audiograms[b] (n=45)
VS patients with serviceable pre-operative hearing (VS-SH cohort)[c] (n=22)
VS-SH males: n=7, VS-SH females: n=15
VS patients with non-serviceable pre-operative hearing (VS-NSH cohort)[d] (n=23)
VS-NSH males: n=15, VS-NSH females: n=8
Class A
Class B
Class C
Class D
VS patients with good pre-operative hearing (VS-GH cohort)[e] (n=13)
VS-GH males: n=6, VS-GH females: n=7
VS patients with poor pre-operative hearing (VS-PH cohort)[f] (n=32)
VS-PH males: n=16, VS-PH females: n=16

Fig. S1. CONSORT flow diagram for the VS cohort

Sample selection flowchart for the VS discovery (A) and validation cohorts (B). Among the overall cohort of eligible VS patients, subgroups were further defined by pre-operative hearing ability based on AAO-HNS guidelines.

Notes: [a] Eligible patients had unilateral, sporadic VS that had not been previously resected or irradiated; [b] Four VS patients had no pre-operative audiograms (discovery cohort) and five VS patients had missing pure tone average or word recognition scores (validation cohort). [c]Serviceable hearing was defined as AAO-HNS Class A and B hearing (PTA ≤ 50 dB and WR score ≥ 50%). [d]Non-serviceable hearing was defined as AAO-HNS Class C and D hearing (either PTA > 50 dB or WRS <50%). [e]GH was defined as word recognition score >70% and pure tone average <30 decibels (dB) (AAO-HNS Class A). [f] PH was defined as word recognition score ≤70% and pure tone average ≥30 dB (AAO-HNS Class B, C and D). Abbreviation: NF2, neurofibromatosis 2.

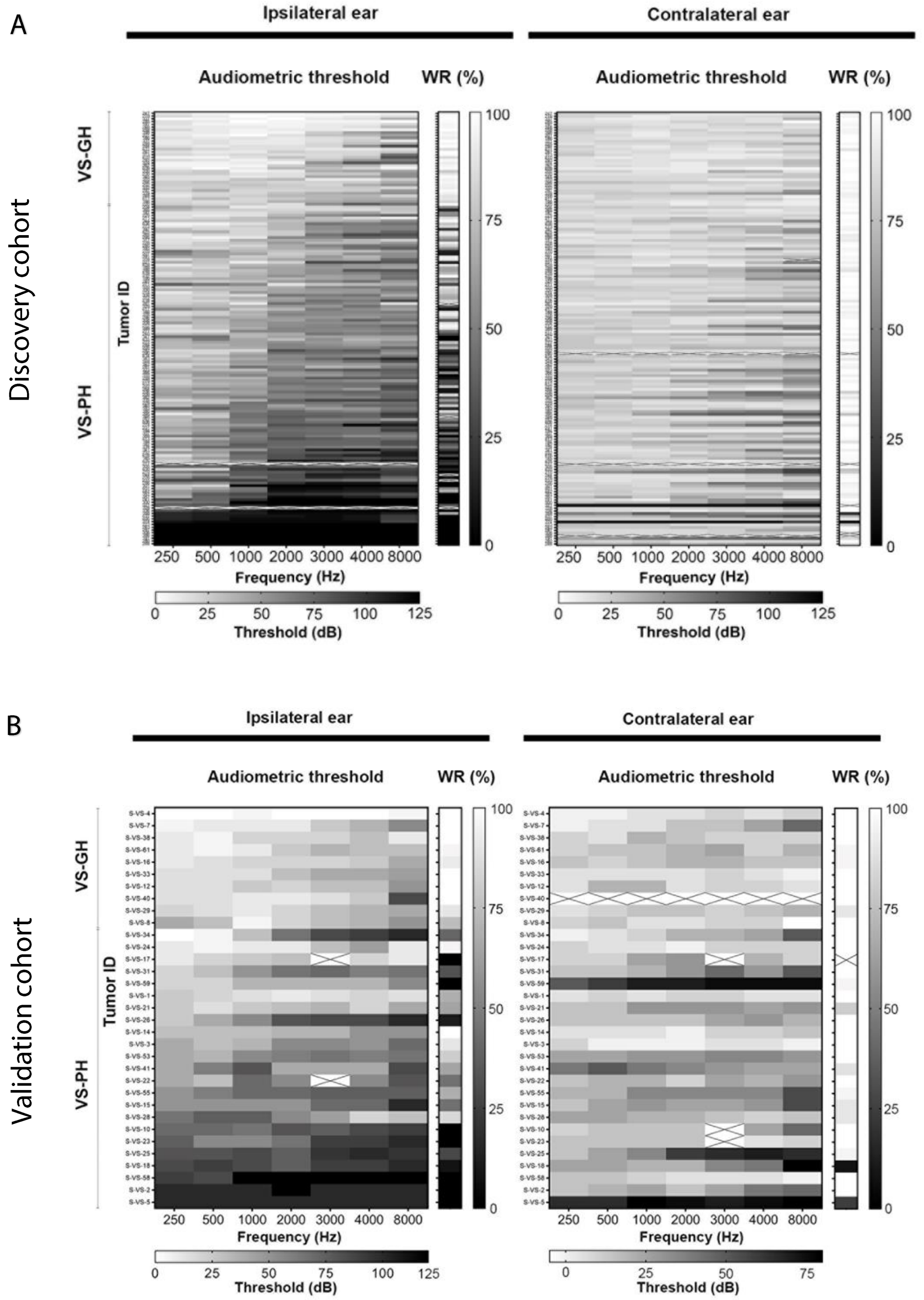

Fig. S2. Hearing characteristics of VS patients in the discovery and validation cohorts

Abbreviation: dB, decibel; GH, good hearing; Hz, hertz; PH, poor hearing; VS, vestibular schwannoma; WR, word recognition.

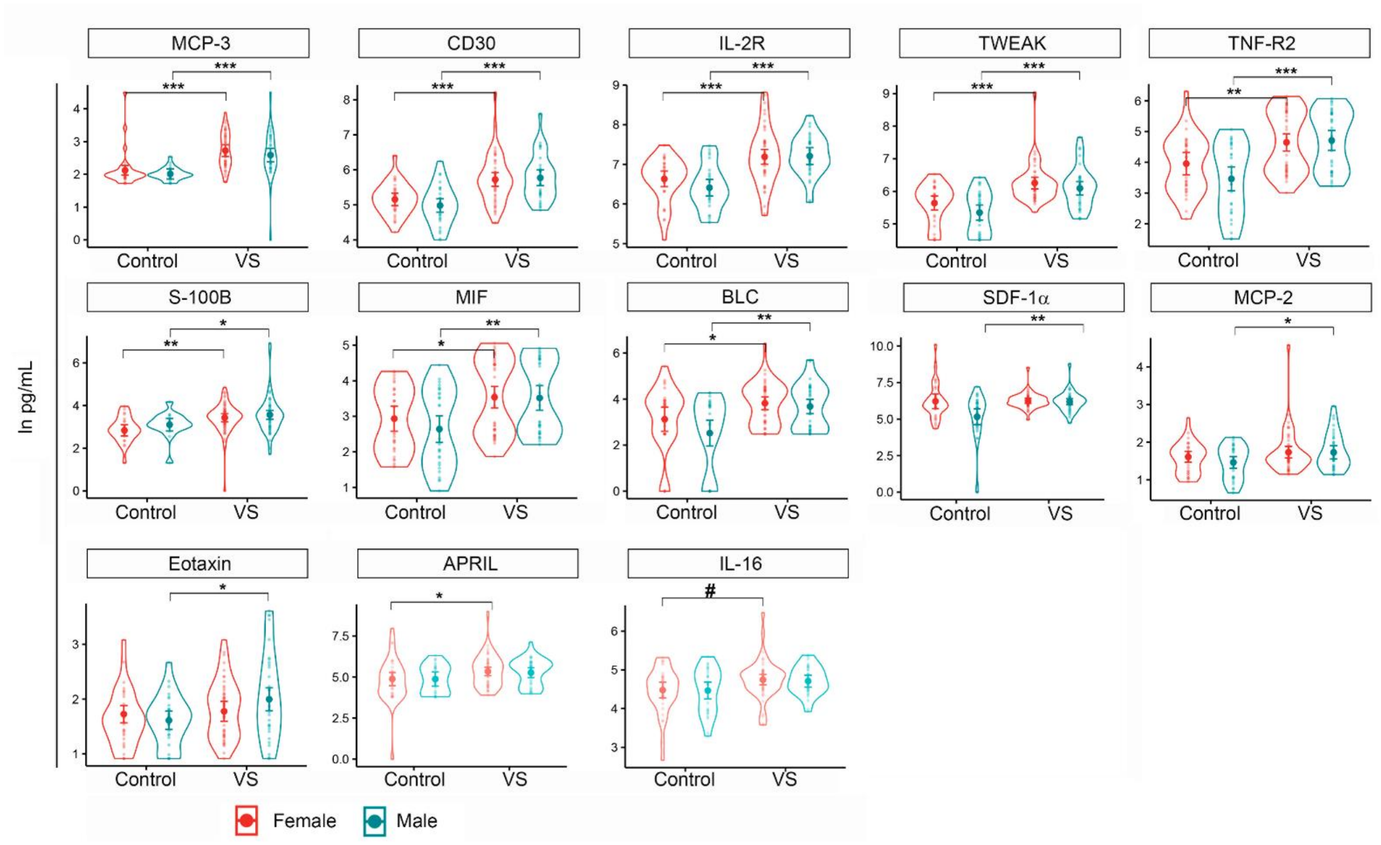

Fig. S3. Significantly elevated candidate biomarkers in female and male VS patients

Abbreviations: VS, vestibular schwannoma. **Padj*<0.05, ***Padj*<0.01, ****Padj*<0.001, #significant at *P*<0.05 prior to adjustment.

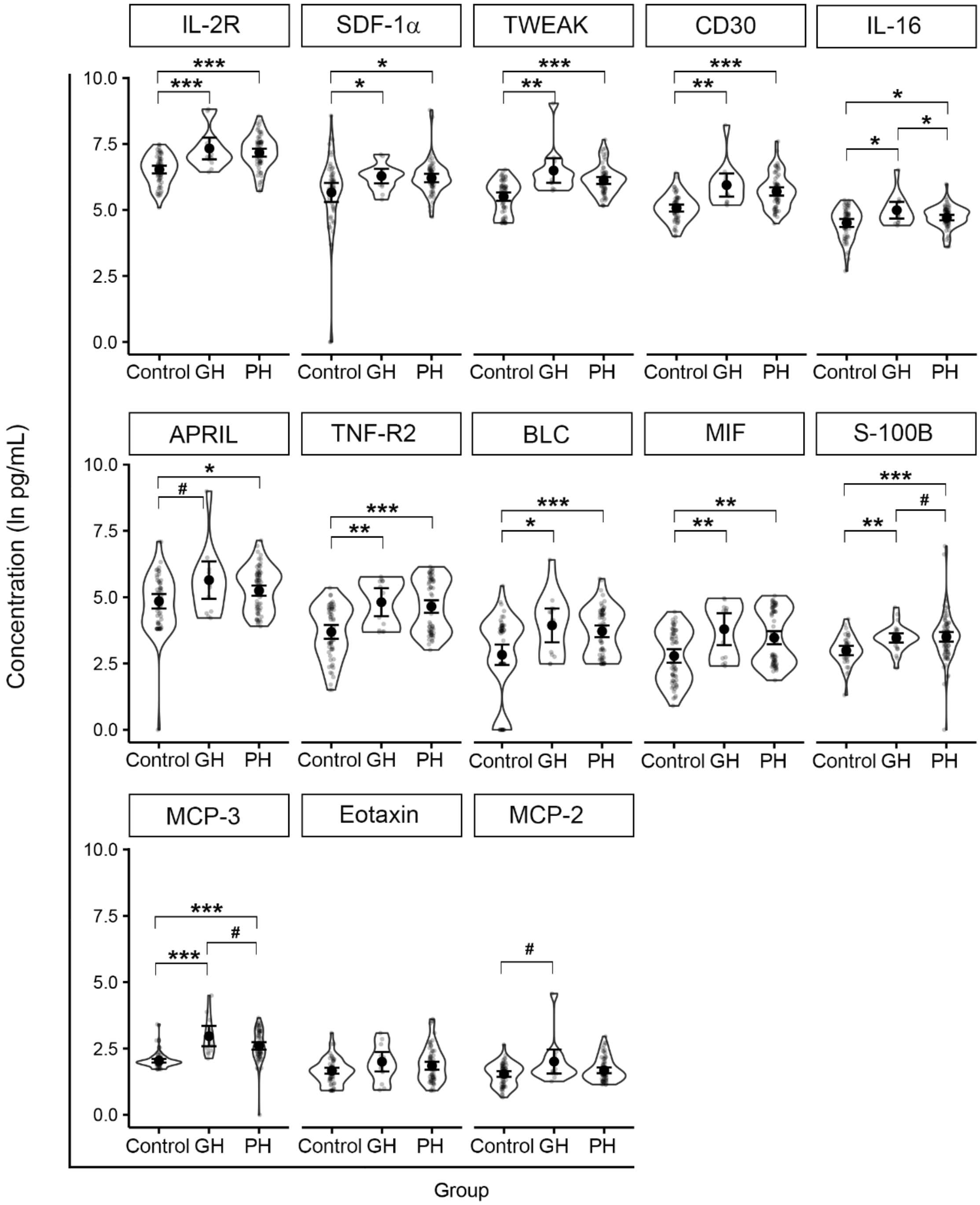

Fig. S4. Plasma levels of candidate biomarkers in VS-GH patients, VS-PH patients, and controls

Abbreviations: GH, good hearing; PH, poor hearing; VS, vestibular schwannoma. **Padj*<0.05, ***Padj*<0.01, ****Padj*<0.001, #significant at *P*<0.05 prior to adjustment.

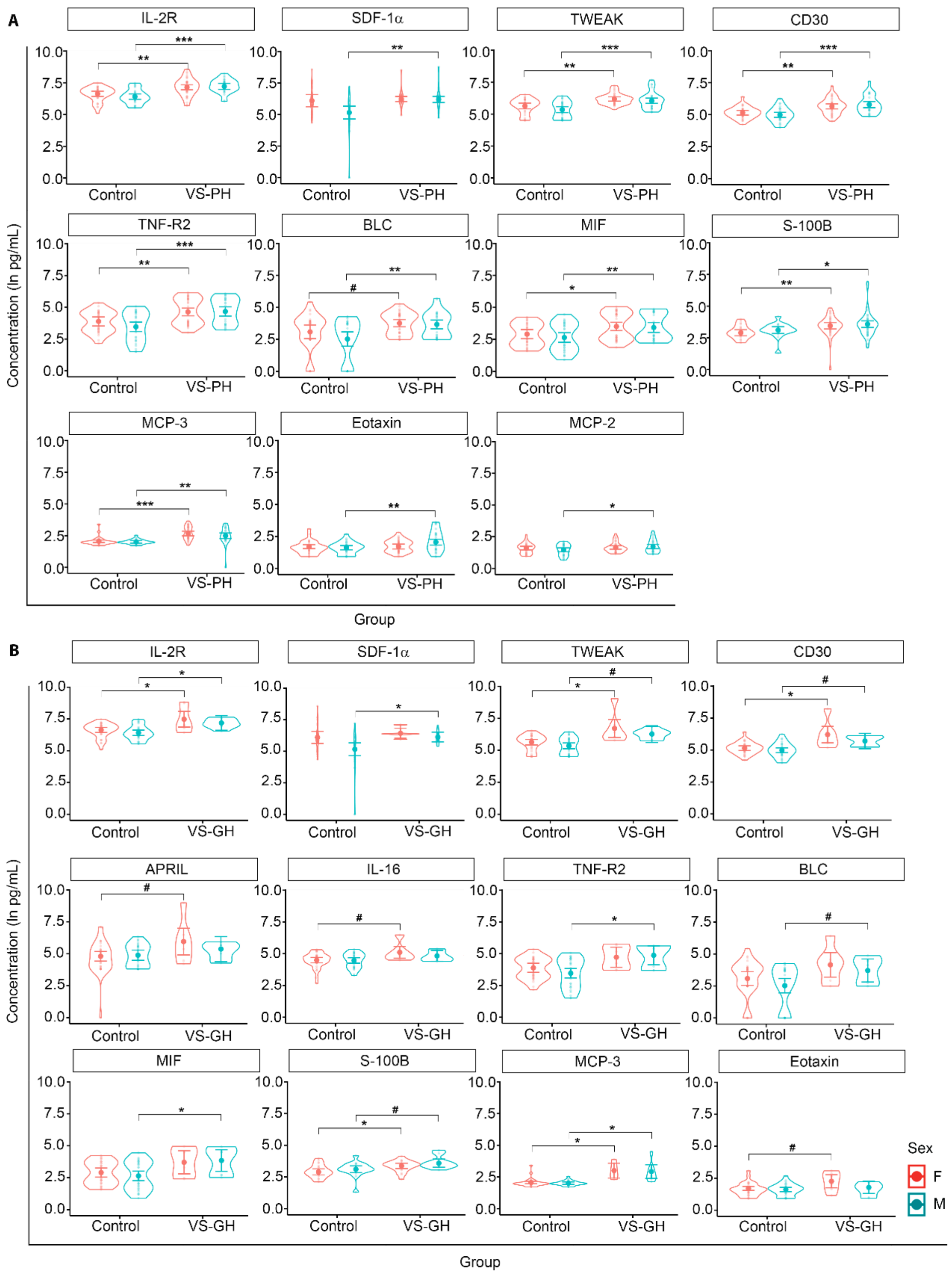

Fig. S5. Significantly elevated candidate biomarkers in female and male VS patients with (A) poor or (B) good hearing compared to controls

Abbreviations: GH, good hearing; PH, poor hearing; VS, vestibular schwannoma. **Padj*<0.05, ***Padj*<0.01, ****Padj*<0.001, #significant at *P*<0.05 prior to adjustment.

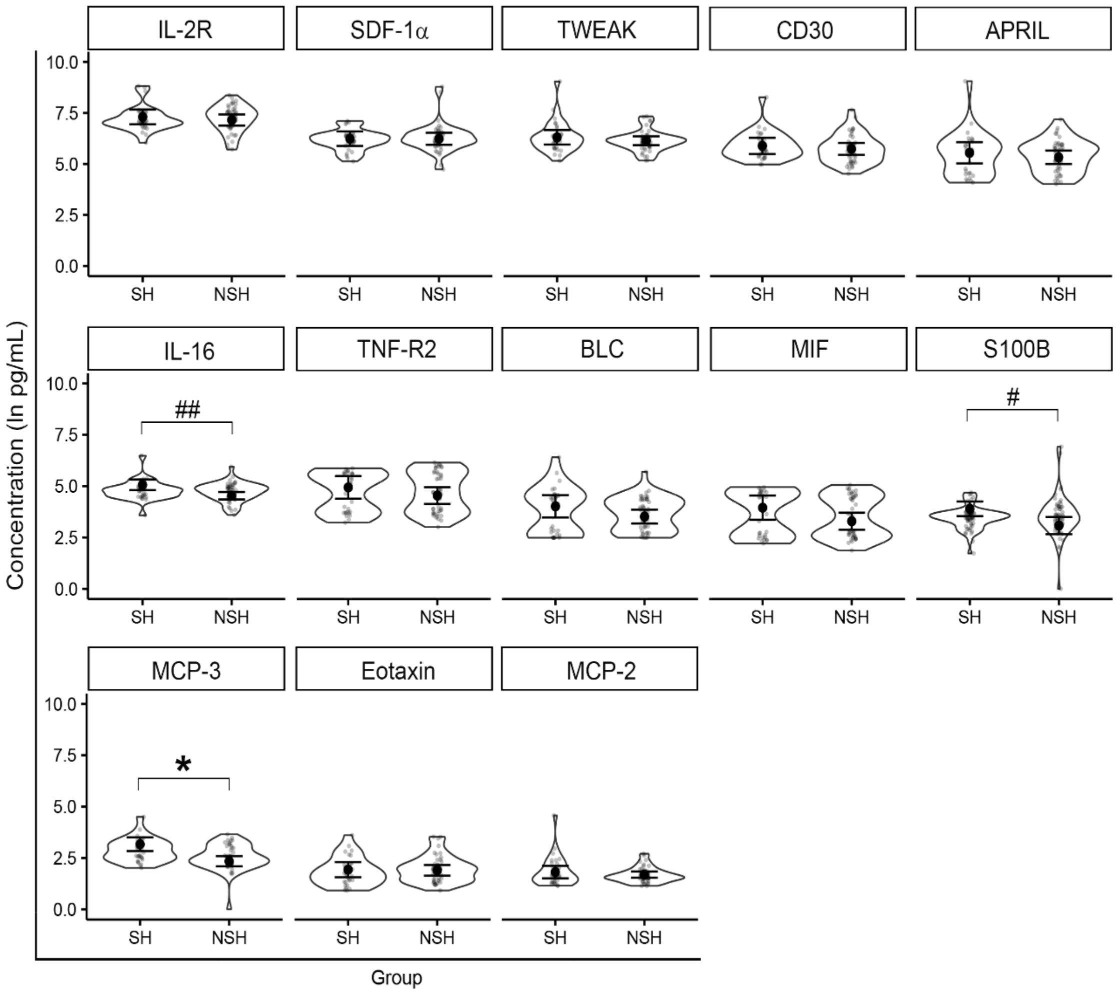

Fig. S6. Plasma levels of candidate biomarkers in VS patients with serviceable and non-serviceable hearing.

The serviceable hearing was defined as AAO-HNS Class A and B hearing (PTA ≤ 50 dB and WRS ≥ 50%). Non-serviceable hearing was defines as AAO-HNS Class C and D hearing (either PTA > 50 dB or WRS <50%). Elevated MCP-3 is associated with serviceable hearing. IL-16 and S100B showed a trend for association with serviceable hearing. *Padj<0.05, #and ## significant at P<0.05 and P<0.001, respectively prior to adjustment. The full names of candidate biomarkers are listed in Table S1. Abbreviations: SH, serviceable hearing; NSH, non-serviceable hearing.

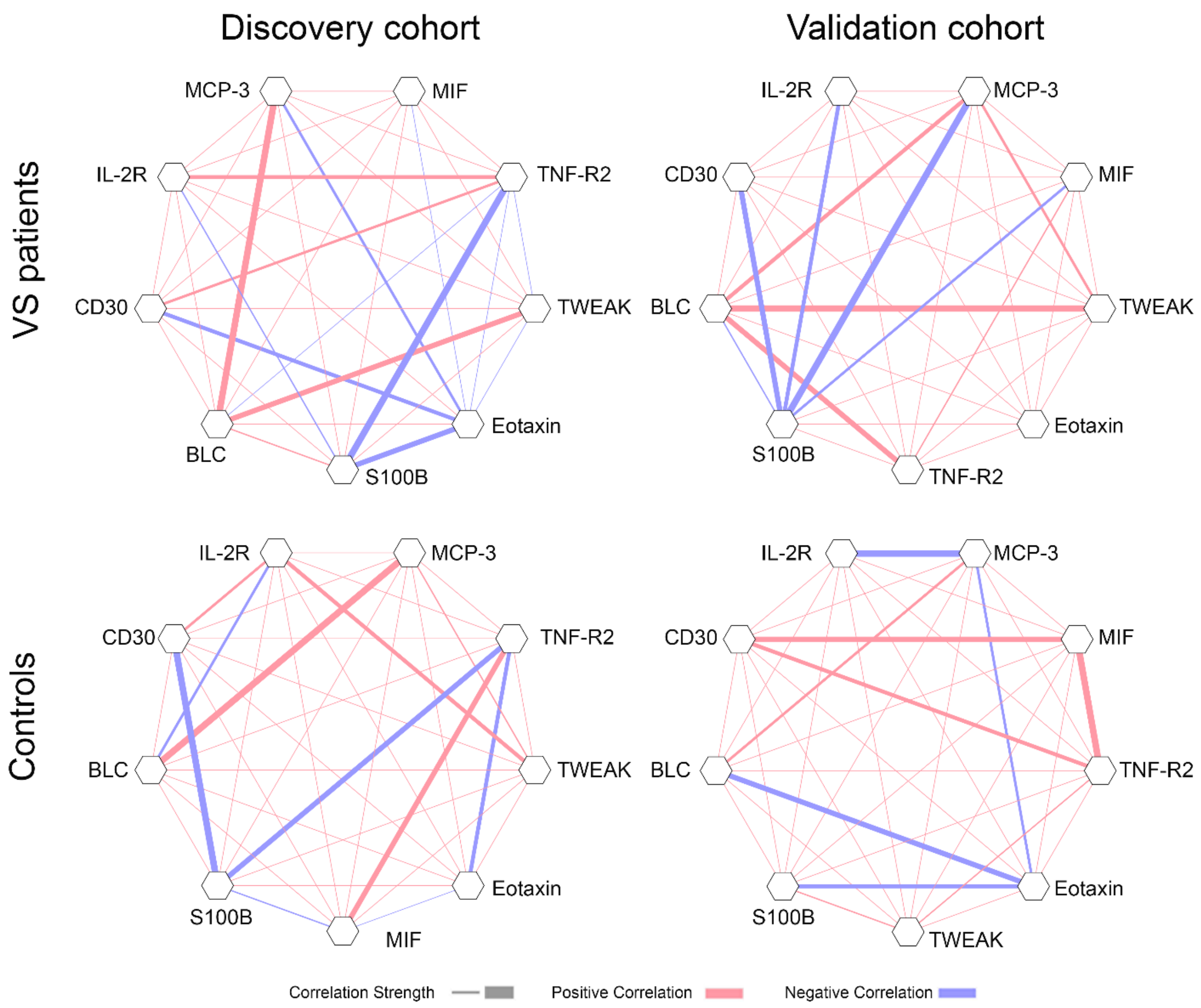

Fig. S7. Correlation networks of candidate biomarkers
Abbreviation: VS, vestibular schwannoma.

Table S1. List and full names of assayed immune factors

Common name	Full name	Common name	Full name	Common name	Full name
APRIL	A proliferation-inducing ligand	IL-1α	Interleukin-1α	MDC	Macrophage-derived chemokine (CCL22)
BAFF	B-cell activating factor	IL-1β	Interleukin-1β	MIF	Macrophage migration inhibitory factor
BLC	B lymphocyte chemoattractant (CXCL13)	IL-2	Interleukin-2	MIG	Monokine induced by interferon gamma (CXCL9)
CD30	CD30	IL-20	Interleukin-20	MIP-1α	Macrophage inflammatory protein-1α (CCL3)
CD40L	CD40 ligand	IL-21	Interleukin-21	MIP-1β	Macrophage inflammatory protein-1β (CCL4)
ENA-78	Epithelial neutrophil-activating peptide 78 (CXCL5)	IL-22	Interleukin-22	MIP-3α	Macrophage inflammatory protein-3α (CCL20)
Eotaxin	Eotaxin (CCL11)	IL-23	Interleukin-23	MMP-1	Matrix metalloproteinase-1
Eotaxin-2	Eotaxin-2 (CCL24)	IL-27	Interleukin-27		
Eotaxin-3	Eotaxin-3 (CCL26)	IL-2R	Interleukin-2R	NGF- β	Nerve Growth Factor-β
FGF-2	Fibroblast Growth Factor-2	IL-3	Interleukin-3	S100B	S100B
Fractalkine	Fractalkine (CX3CL1)	IL-31	Interleukin-31	SCF	Stem cell factor
G-CSF	Granulocyte-colony stimulating factor	IL-4	Interleukin-4	SDF-1α	Stromal cell-derived factor 1α (CXCL12)
GM-CSF	Granulocyte macrophage colony-stimulating factor	IL-5	Interleukin-5	TNF-α	Tumor necrosis factor- α
GRO-α	Growth-related oncogene α (CXCL1)	IL-6	Interleukin-6	TNF- β	Tumor necrosis factor- β
HGF	Hepatocyte Growth Factor	IL-7	Interleukin-7	TNF-R2	Tumor necrosis factor- receptor 2
IFN-α	Interferon-α	IL-8	Interleukin-8	TRAIL	Tumor necrosis factor (TNF)-related apoptosis-inducing ligand
IFN-g	Interferon-ɣ	IL-9	Interleukin-9	TSLP	Thymic stromal lymphopoietin
IL-10	Interleukin-10	IP-10	Interferon-γ inducible protein-10 (CXCL10)	TWEAK	Tumor necrosis factor-like weak inducer of apoptosis
IL-12p70	Interleukin-12p70	I-TAC	Interferon–inducible T Cell Alpha	VEGF-A	Vascular endothelial growth factor-A

			Chemoattractant (CXCL11)
IL-13	Interleukin-13	LIF	Leukemia inhibitory factor
IL-15	Interleukin-15	MCP-1	Monocyte chemoattractant protein-1 (CCL2)
IL-16	Interleukin-16	MCP-2	Monocyte chemoattractant protein-2 (CCL8)
IL-17A	Interleukin-17A	MCP-3	Monocyte chemoattractant protein-3 (CCL7)
IL-18	Interleukin-18	M-CSF	Macrophage colony-stimulating factor

Table S2. Detectability of candidate biomarkers in plasma of VS patients

Candidate biomarkers	Samples between LLOQ and ULOQ	Candidate biomarkers	Samples between LLOQ and ULOQ	Candidate biomarkers	Samples between LLOQ and ULOQ
**APRIL**	**81/81 (100%) [a]**	**IL-18**	**123/123 (100%) [b]**	M-CSF	8/81 (10%) [a]
BAFF	56/81 (69%) [a]	IL-1α	8/81 (10%) [a]	**MDC**	**77/81 (95%) [a]**
**BLC**	**73/81 (90%) [a]**	IL-1β	6/81 (7%) [a]	**MIF**	**81/81 (100%) [a]**
**CD30**	**81/81 (100%) [a]**	IL-2	49/81 (60%) [a]	MIG	30/81 (37%) [a]
CD40L	32/81(40%) [a]	IL-20	19/81 (23%) [a]	MIP-1α	43/81 (53%) [a]
ENA-78	49/81 (60%) [a]	IL-21	7/81 (9%) [a]	MIP-1β	36/81 (44%) [a]
**Eotaxin**	**77/81 (95%) [a]**	IL-22	6/81 (7%) [a]	MIP-3α	5/81 (6%) [a]
**Eotaxin-2**	**80/81 (99%) [a]**	IL-23	14/81 (17%) [a]	MMP1	7/81 (9%) [a]
Eotaxin-3	47/81 (58%) [a]	IL-27	16/81 (20%) [a]	NGF- β	10/81 (12%) [a]
FGF-2	35/81 (43%) [a]	**IL-2R**	**81/81 (100%) [a]**	**S100 B**	**122/123 (99%) [c]**
Fractalkine	10/81 (12%) [a]	IL-3	4/81 (5%) [a]	SCF	19/81 (23%) [a]
G-CSF	6/81 (7%) [a]	IL-31	5/81 (6%) [a]	**SDF-1α**	**81/81 (100%) [a]**
GM-CSF	2/81 (2%) [a]	IL-4	15/81 (19%) [a]	TNF-α	3/81 (4%) [a]
GRO-α	1/81 (1%) [a]	IL-5	7/81 (9%) [a]	TNF-α	29/123 (24%) [b]
**HGF**	**80/81 (99%) [a]**	IL-6	8/81 (10%) [a]	TNF- β	14/81 (17%) [a]
IFN-α	5/81 (6%) [a]	IL-7	14/81 (17%) [a]	**TNF-R2**	**81/81 (100%) [a]**
IFN-γ	21/81 (26%) [a]	IL-8	15/81 (19%) [a]	TRAIL	49/81 (60%) [a]
IL-10	16/81 (20%) [a]	IL-9	5/81 (6%) [a]	TSLP	15/81 (19%) [a]
IL-12p70	4/81 (5%) [a]	**IP-10**	**80/81 (99%) [a]**	**TWEAK**	**81/81 (100%) [a]**
IL-13	18/81 (22%) [a]	I-TAC	5/81 (6%) [a]	**VEGF-A**	**76/81 (94%) [a]**
IL-15	13/81 (16%) [a]	LIF	18/81 (22%) [a]		
**IL-16**	**81/81 (100%) [a]**	**MCP-1**	**81/81 (100%) [a]**		
IL-17A	33/81 (41%) [a]	**MCP-2**	**80/81 (99%) [a]**		
IL-18	43/81 (53%) [a]	**MCP-3**	**79/81 (98%) [a]**		

Candidate biomarkers with an absolute concentration between the LLOQ and ULOQ calculated in 75% or more tested plasma samples were included in the study. Twenty of 66 tested candidate biomarkers fulfilled the inclusion criteria (bold). Included candidate biomarkers whose absolute concentration was between 0 pg/mL and LLOQ were assigned the value of the corresponding LLOQ. Candidate biomarkers exceeding ULOQ were approximated with the corresponding ULOQ. Abbreviations: LLOQ, lower limit of quantification; ULOQ, upper limit of quantification; VS, vestibular schwannoma. Notes: [a] Assessed with Luminex assay; [b] Assessed with electrochemiluminescence assay; [c] Assessed with ELISA assay.

Table S3. The diagnostic power of candidate biomarker panels containing MCP-3 and S100B assessed in the discovery cohort (CombiROC combinatorial analysis)

Biomarker combinations	Symbol	AUC	SE	SP	Opt Cutoff
MCP3-S100B-TNFR2-IL2R-BLC-TWEAK	Combo CDXXIV	1	1	1	0.5
MCP3-S100B-TNFR2-MIF-IL2R-BLC-TWEAK	Combo CDLXXIII	1	1	1	0.5
MCP3-S100B-TNFR2-MIF-IL2R-BLC-Eotaxin	Combo CDLXXIV	1	1	1	0.5
MCP3-S100B-TNFR2-CD30-IL2R-BLC-TWEAK	Combo CDLXXIX	1	1	1	0.5
MCP3-S100B-TNFR2-IL2R-BLC-TWEAK-Eotaxin	Combo CDLXXXIV	1	1	1	0.5
MCP3-S100B-TNFR2-MIF-CD30-IL2R-BLC-TWEAK	Combo CDXCIV	1	1	1	0.5
MCP3-S100B-TNFR2-MIF-CD30-IL2R-BLC-Eotaxin	Combo CDXCV	1	1	1	0.5
MCP3-S100B-TNFR2-MIF-CD30-IL2R-TWEAK-Eotaxin	Combo CDXCVI	1	1	1	0.5
MCP3-S100B-TNFR2-MIF-IL2R-BLC-TWEAK-Eotaxin	Combo CDXCIX	1	1	1	0.5
MCP3-S100B-TNFR2-CD30-IL2R-BLC-TWEAK-Eotaxin	Combo D	1	1	1	0.5
**MCP3-S100B-TNFR2-MIF-CD30-IL2R-BLC-TWEAK-Eotaxin**	**Combo DII**	**1**	**1**	**1**	**0.5**
MCP3-S100B-TNFR2-MIF-IL2R-Eotaxin	Combo CCCXCVIII	0.991	1	0.958	0.331
MCP3-S100B-TNFR2-MIF-CD30-IL2R-Eotaxin	Combo CDLXII	0.991	1	0.958	0.362
MCP3-S100B-TNFR2-MIF-IL2R-TWEAK-Eotaxin	Combo CDLXXV	0.991	1	0.958	0.284
MCP3-S100B-TNFR2-CD30-IL2R-BLC-Eotaxin	Combo CDLXXX	0.991	0.958	0.958	0.61
MCP3-S100B-TNFR2-MIF-CD30-BLC-TWEAK-Eotaxin	Combo CDXCVII	0.986	0.958	0.958	0.456
MCP3-S100B-TNFR2-IL2R-BLC-Eotaxin	Combo CDXXV	0.984	0.958	0.958	0.636
MCP3-S100B-TNFR2-CD30-IL2R-TWEAK-Eotaxin	Combo CDLXXXI	0.984	0.958	0.958	0.684
MCP3-S100B-TNFR2-MIF-IL2R-TWEAK	Combo CCCXCVII	0.983	0.958	0.917	0.295
MCP3-S100B-TNFR2-CD30-IL2R-Eotaxin	Combo CDXIII	0.983	0.958	0.958	0.612
MCP3-S100B-TNFR2-IL2R-TWEAK-Eotaxin	Combo CDXXVI	0.981	0.958	0.958	0.619
MCP3-S100B-TNFR2-BLC-TWEAK-Eotaxin	Combo CDXXVII	0.981	0.917	0.958	0.497
MCP3-S100B-TNFR2-MIF-CD30-IL2R-TWEAK	Combo CDLXI	0.981	0.958	0.917	0.29
MCP3-S100B-TNFR2-MIF-CD30-TWEAK-Eotaxin	Combo CDLXVI	0.981	1	0.833	0.186
MCP3-S100B-TNFR2-MIF-BLC-TWEAK-Eotaxin	Combo CDLXXVI	0.981	1	0.875	0.331
MCP3-S100B-MIF-CD30-IL2R-BLC-TWEAK	Combo CDLXXXVI	0.981	0.958	0.917	0.473
MCP3-S100B-MIF-IL2R-BLC-TWEAK-Eotaxin	Combo CDXCI	0.981	0.958	0.917	0.365
MCP3-S100B-MIF-CD30-IL2R-BLC-TWEAK-Eotaxin	Combo DI	0.981	0.958	0.875	0.382
MCP3-S100B-TNFR2-MIF-CD30-IL2R	Combo CCCLXXIV	0.979	0.917	0.958	0.663
MCP3-S100B-TNFR2-MIF-CD30-Eotaxin	Combo CCCLXXXII	0.979	1	0.833	0.207

MCP3-S100B-TNFR2-MIF-CD30-IL2R-BLC	Combo CDLIX	0.979	0.958	0.917	0.364
MCP3-S100B-TNFR2-MIF-CD30-BLC-TWEAK	Combo CDLXIV	0.979	0.958	0.917	0.356
MCP3-S100B-TNFR2-CD30-BLC-TWEAK-Eotaxin	Combo CDLXXXII	0.979	0.917	0.958	0.561
MCP3-S100B-TNFR2-IL2R-Eotaxin	Combo CCCVII	0.977	0.958	0.958	0.591
MCP3-S100B-TNFR2-MIF-IL2R-BLC	Combo CCCXCV	0.977	0.958	0.917	0.347
MCP3-S100B-TNFR2-MIF-BLC-Eotaxin	Combo CDI	0.977	1	0.833	0.172
MCP3-S100B-TNFR2-MIF-TWEAK-Eotaxin	Combo CDII	0.977	0.833	1	0.843
MCP3-S100B-TNFR2-MIF-Eotaxin	Combo CCLXXI	0.976	0.833	1	0.866
MCP3-S100B-TNFR2-CD30-Eotaxin	Combo CCXCI	0.976	0.958	0.875	0.33
MCP3-S100B-TNFR2-MIF-CD30-BLC-Eotaxin	Combo CDLXV	0.976	1	0.833	0.172
MCP3-S100B-TNFR2-Eotaxin	Combo CLXVI	0.974	0.958	0.875	0.328
MCP3-S100B-TNFR2-MIF-BLC-TWEAK	Combo CD	0.974	0.917	0.958	0.493
MCP3-S100B-TNFR2-CD30-BLC-Eotaxin	Combo CDXVI	0.974	0.875	0.958	0.72
MCP3-S100B-MIF-IL2R-BLC-TWEAK	Combo CDXLV	0.974	0.917	0.958	0.525
MCP3-S100B-TNFR2-MIF-IL2R	Combo CCLXV	0.972	0.917	0.958	0.602
MCP3-S100B-TNFR2-BLC-Eotaxin	Combo CCCX	0.972	0.875	0.958	0.696
MCP3-S100B-TNFR2-TWEAK-Eotaxin	Combo CCCXI	0.972	0.875	0.958	0.7
MCP3-S100B-TNFR2-CD30-IL2R-BLC	Combo CDX	0.972	0.958	0.958	0.378
MCP3-S100B-TNFR2-CD30-BLC-TWEAK	Combo CDXV	0.972	0.917	0.917	0.521
MCP3-S100B-TNFR2-CD30-TWEAK-Eotaxin	Combo CDXVII	0.972	0.875	0.958	0.699
MCP3-S100B-MIF-BLC-TWEAK-Eotaxin	Combo CDXLVIII	0.972	0.958	0.917	0.373
MCP3-S100B-MIF-CD30-BLC-TWEAK-Eotaxin	Combo CDLXXXIX	0.972	0.958	0.875	0.308
MCP3-S100B-TNFR2-IL2R-BLC	Combo CCCIV	0.97	0.958	0.958	0.397
MCP3-S100B-TNFR2-IL2R-TWEAK	Combo CCCVI	0.97	0.958	0.958	0.394
MCP3-S100B-TNFR2-MIF	Combo CXXXI	0.969	0.958	0.875	0.198
MCP3-S100B-TNFR2-BLC	Combo CLXIII	0.969	0.958	0.917	0.392
MCP3-S100B-TNFR2-MIF-TWEAK	Combo CCLXX	0.969	0.958	0.875	0.194
MCP3-S100B-TNFR2-BLC-TWEAK	Combo CCCIX	0.969	0.958	0.833	0.21
MCP3-S100B-TNFR2-MIF-CD30-TWEAK	Combo CCCLXXXI	0.969	0.958	0.875	0.191
MCP3-S100B-TNFR2-CD30-IL2R-TWEAK	Combo CDXII	0.969	0.958	0.958	0.404
MCP3-S100B-MIF-CD30-BLC-TWEAK	Combo CDXXXVI	0.969	0.958	0.875	0.333
MCP3-S100B-TNFR2CD30	Combo CXLVI	0.967	0.958	0.917	0.331
MCP3-S100B-TNFR2-TWEAK	Combo CLXV	0.967	0.958	0.917	0.308
MCP3-S100B-TNFR2-MIF-CD30	Combo CCLI	0.967	0.958	0.875	0.199
MCP3-S100B-TNFR2-CD30-TWEAK	Combo CCXC	0.967	0.958	0.917	0.329
MCP3-S100B-MIF-IL2R-BLC-Eotaxin	Combo CDXLVI	0.967	0.875	1	0.748

MCP3-S100B-MIF-IL2R-TWEAK-Eotaxin	Combo CDXLVII	0.967	0.917	0.958	0.523
MCP3-S100B-TNFR2	Combo LIV	0.965	0.958	0.917	0.326
MCP3-S100B-TNFR2-MIF-BLC	Combo CCLXVIII	0.965	0.958	0.875	0.231
MCP3-S100B-TNFR2-CD30IL2R	Combo CCLXXXV	0.965	0.958	0.958	0.381
MCP3-S100B-TNFR2-CD30-BLC	Combo CCLXXXVIII	0.965	0.958	0.917	0.392
MCP3-S100B-TNFR2-MIF-CD30-BLC	Combo CCCLXXIX	0.965	0.958	0.875	0.235
MCP3-S100B-MIF-CD30-IL2R-TWEAK-Eotaxin	Combo CDLXXXVIII	0.965	0.917	0.958	0.49
MCP3-S100B-MIF-IL2R-TWEAK	Combo CCCXLI	0.964	0.875	1	0.633
MCP3-S100B-MIF-BLC-TWEAK	Combo CCCXLIV	0.964	0.875	0.958	0.651
MCP3-S100B-MIF-CD30-IL2R-BLC-Eotaxin	Combo CDLXXXVII	0.964	0.875	1	0.749
MCP3-S100B-MIF-CD30-IL2R-TWEAK	Combo CDXXXIII	0.962	0.875	1	0.618
MCP3-S100B-TNFR2-IL2R	Combo CLX	0.96	0.958	0.958	0.414
MCP3-S100B-MIF-BLC-Eotaxin	Combo CCCXLV	0.96	0.875	0.958	0.683
MCP3-S100B-MIF-IL2R-Eotaxin	Combo CCCXLII	0.958	0.875	1	0.745
MCP3-S100B-MIF-CD30-BLC-Eotaxin	Combo CDXXXVII	0.957	0.875	0.958	0.696
MCP3-S100B-MIF-TWEAK	Combo CC	0.955	0.833	1	0.722
MCP3-S100B-MIF-Eotaxin	Combo CCI	0.955	0.875	0.958	0.599
MCP3-S100B-MIF-CD30-TWEAK	Combo CCCXXV	0.955	0.833	1	0.723
MCP3-S100B-MIF-CD30-IL2R-Eotaxin	Combo CDXXXIV	0.955	0.875	1	0.777
MCP3-S100B-MIF-IL2R	Combo CXCV	0.953	0.875	1	0.676
MCP3-S100B-MIF-BLC	Combo CXCVIII	0.953	0.875	1	0.739
MCP3-S100B-MIF-CD30-BLC	Combo CCCXXIII	0.953	0.875	1	0.748
MCP3-S100B-MIF-TWEAK-Eotaxin	Combo CCCXLVI	0.953	0.833	1	0.753
MCP3-S100B-MIF-CD30-IL2R-BLC	Combo CDXXXI	0.953	0.875	0.958	0.66
MCP3-S100B-MIF-CD30-TWEAK-Eotaxin	Combo CDXXXVIII	0.953	0.833	1	0.754
MCP3-S100B-MIF-CD30-Eotaxin	Combo CCCXXVI	0.951	0.833	1	0.768
MCP3-S100B-MIF-CD30IL2R	Combo CCCXX	0.95	0.875	1	0.639
MCP3-S100B-MIF	Combo LXXV	0.948	0.875	0.958	0.633
MCP3-S100B-MIFCD30	Combo CLXXXI	0.946	0.875	1	0.662
MCP3-S100B-CD30-IL2R-Eotaxin	Combo CCCLVII	0.938	0.917	0.833	0.431
MCP3-S100B-CD30-BLC-TWEAK-Eotaxin	Combo CDLIV	0.938	0.833	0.958	0.716
MCP3-S100B-MIF-IL2R-BLC	Combo CCCXXIX	0.936	0.875	0.958	0.583
MCP3-S100B-CD30-IL2R-TWEAK-Eotaxin	Combo CDLIII	0.936	0.917	0.833	0.426
MCP3-S100B-CD30-IL2R-BLC-TWEAK-Eotaxin	Combo CDXCII	0.934	0.833	0.958	0.676
MCP3-S100B-IL2R-BLC-TWEAK-Eotaxin	Combo CDLVI	0.932	0.833	0.958	0.66
MCP3-S100B-IL2R-BLC-Eotaxin	Combo CCCLXIX	0.931	1	0.75	0.228
MCP3-S100B-CD30-IL2R-BLC-Eotaxin	Combo CDLII	0.931	0.917	0.833	0.426
MCP3-S100B-CD30-Eotaxin	Combo CCXXI	0.929	0.875	0.917	0.505

MCP3-S100B-CD30-BLC-Eotaxin	Combo CCCLX	0.929	0.875	0.917	0.524
MCP3-S100B-CD30-IL2R-BLC	Combo CCCLIV	0.927	0.917	0.833	0.388
MCP3-S100B-CD30-TWEAK-Eotaxin	Combo CCCLXI	0.927	0.875	0.917	0.488
MCP3-S100B-BLC-TWEAK-Eotaxin	Combo CCCLXXI	0.927	0.875	0.958	0.557
MCP3-S100B-IL2R-Eotaxin	Combo CCXXXVII	0.925	0.875	0.875	0.461
MCP3-S100B-TWEAK-Eotaxin	Combo CCXLI	0.924	1	0.708	0.193
MCP3-S100B-CD30-BLC-TWEAK	Combo CCCLIX	0.924	0.875	0.875	0.485
MCP3-S100B-IL2R-TWEAK-Eotaxin	Combo CCCLXX	0.924	0.875	0.875	0.458
MCP3-S100B-CD30-BLC	Combo CCXVIII	0.92	0.833	0.958	0.57
MCP3-S100B-BLC-Eotaxin	Combo CCXL	0.92	0.958	0.792	0.314
MCP3-S100B-CD30-IL2R-BLC-TWEAK	Combo CDLI	0.92	0.875	0.875	0.359
MCP3-S100B-Eotaxin	Combo CX	0.918	1	0.708	0.183
MCP3-S100B-CD30-IL2R	Combo CCXV	0.918	0.833	0.917	0.529
MCP3-S100B-CD30-IL2R-TWEAK	Combo CCCLVI	0.918	0.833	0.917	0.528
MCP3-S100B-CD30	Combo XC	0.913	0.833	0.917	0.541
MCP3-S100B-CD30-TWEAK	Combo CCXX	0.913	0.833	0.917	0.525
MCP3-S100B-IL2R-BLC-TWEAK	Combo CCCLXVIII	0.913	0.833	0.958	0.64
MCP3-S100B-IL2R-BLC	Combo CCXXXIV	0.91	0.833	0.917	0.578
MCP3-S100B-IL2R-TWEAK	Combo CCXXXVI	0.91	0.833	0.917	0.554
MCP3-S100B-BLC-TWEAK	Combo CCXXXIX	0.908	0.833	0.958	0.52
MCP3-S100B-IL2R	Combo CIV	0.906	0.833	0.917	0.54
MCP3-S100B-TWEAK	Combo CIX	0.884	0.792	0.917	0.553
MCP3-S100B-BLC	Combo CVII	0.882	0.917	0.833	0.31
MCP3-S100B	Combo XXVI	0.88	0.917	0.833	0.282

9-biomarker panel (combo DII) is in bold text. Abbreviations: AUC, area under curve; Opt, optimal; SE, sensitivity; SP, specificity.

Table S4. The diagnostic power of candidate biomarker panels containing MCP-3 and S100B assessed in the validation cohort (CombiROC combinatorial analysis)

Biomarker combinations	Symbol	AUC	SE	SP	Opt Cutoff
MCP3-S100B-TNFR2-MIF-CD30-IL2R-BLC-Eotaxin	Combo CDXCV	0.879	0.938	0.688	0.312
MCP3-S100B-TNFR2-MIF-CD30-IL2R-Eotaxin	Combo CDLXII	0.878	0.938	0.688	0.312
MCP3-S100B-TNFR2-MIF-CD30-IL2R-BLC-TWEAK	Combo CDXCIV	0.876	0.875	0.844	0.434
MCP3-S100B-MIF-IL2R-BLC-TWEAK-Eotaxin	Combo CDXCI	0.875	0.812	0.844	0.429
**MCP3-S100B-TNFR2-MIF-CD30-IL2R-BLC-TWEAK-Eotaxin**	**Combo DII**	**0.874**	**0.906**	**0.75**	**0.384**
MCP3-S100B-CD30-IL2R-BLC-TWEAK-Eotaxin	Combo CDXCII	0.874	0.844	0.844	0.445
MCP3-S100B-TNFR2-MIF-CD30-IL2R-TWEAK-Eotaxin	Combo CDXCVI	0.874	0.906	0.75	0.392
MCP3-S100B-TNFR2-CD30-IL2R-BLC-TWEAK-Eotaxin	Combo D	0.873	0.844	0.812	0.439
MCP3-S100B-IL2R-BLC-TWEAK-Eotaxin	Combo CDLVI	0.873	0.781	0.844	0.444
MCP3-S100B-TNFR2-MIF-IL2R-BLC-Eotaxin	Combo CDLXXIV	0.873	0.969	0.656	0.274
MCP3-S100B-TNFR2-MIF-IL2R-TWEAK-Eotaxin	Combo CDLXXV	0.872	0.906	0.719	0.379
MCP3-S100B-MIF-IL2R-TWEAK-Eotaxin	Combo CDXLVII	0.872	0.812	0.812	0.433
MCP3-S100B-TNFR2-IL2R-TWEAK-Eotaxin	Combo CDXXVI	0.872	0.781	0.844	0.447
MCP3-S100B-IL2R-TWEAK-Eotaxin	Combo CCCLXX	0.872	0.781	0.844	0.446
MCP3-S100B-TNFR2-CD30-IL2R-BLC-TWEAK	Combo CDLXXIX	0.872	0.875	0.844	0.447
MCP3-S100B-MIF-CD30-IL2R-BLC-TWEAK-Eotaxin	Combo DI	0.871	0.844	0.844	0.431
MCP3-S100B-MIF-CD30-IL2R-TWEAK-Eotaxin	Combo CDLXXXVIII	0.87	0.844	0.812	0.425
MCP3-S100B-MIF-IL2R-BLC-Eotaxin	Combo CDXLVI	0.87	0.719	0.906	0.609
MCP3-S100B-MIF-IL2R-BLC-TWEAK	Combo CDXLV	0.87	0.781	0.906	0.597
MCP3-S100B-TNFR2-MIF-CD30-BLC-TWEAK	Combo CDLXIV	0.87	0.875	0.844	0.483
MCP3-S100B-TNFR2-MIF-CD30-IL2R-TWEAK	Combo CDLXI	0.87	0.875	0.812	0.436
MCP3-S100B-TNFR2-MIF-CD30-IL2R-BLC	Combo CDLIX	0.87	0.906	0.812	0.42
MCP3-S100B-TNFR2-MIF-IL2R-BLC-TWEAK-Eotaxin	Combo CDXCIX	0.869	0.906	0.688	0.358
MCP3-S100B-TNFR2-IL2R-BLC-TWEAK-Eotaxin	Combo CDLXXXIV	0.869	0.781	0.844	0.444
MCP3-S100B-TNFR2-CD30-IL2R-TWEAK-Eotaxin	Combo CDLXXXI	0.869	0.844	0.812	0.42
MCP3-S100B-CD30-IL2R-TWEAK-Eotaxin	Combo CDLIII	0.869	0.844	0.812	0.421
MCP3-S100B-MIF-CD30-IL2R-BLC-Eotaxin	Combo CDLXXXVII	0.869	0.781	0.844	0.485
MCP3-S100B-TNFR2-MIF-IL2R-Eotaxin	Combo CCCXCVIII	0.869	0.719	0.906	0.61
MCP3-S100B-IL2R-Eotaxin	Combo CCXXXVII	0.869	0.719	0.906	0.613
MCP3-S100B-TNFR2-IL2R-TWEAK	Combo CCCVI	0.869	0.781	0.875	0.588
MCP3-S100B-CD30-IL2R-BLC-Eotaxin	Combo CDLII	0.868	0.906	0.688	0.335

MCP3-S100B-MIF-CD30-IL2R-Eotaxin	Combo CDXXXIV	0.868	0.781	0.844	0.499
MCP3-S100B-MIF-IL2R-Eotaxin	Combo CCCXLII	0.868	0.719	0.906	0.616
MCP3-S100B-TNFR2-IL2R-BLC-TWEAK	Combo CDXXIV	0.868	0.781	0.906	0.605
MCP3-S100B-MIF-IL2R-TWEAK	Combo CCCXLI	0.868	0.875	0.75	0.371
MCP3-S100B-TNFR2-MIF-CD30-BLC	Combo CCCLXXIX	0.868	0.906	0.812	0.467
MCP3-S100B-TNFR2-CD30-IL2R-BLC-Eotaxin	Combo CDLXXX	0.867	0.906	0.719	0.359
MCP3-S100B-MIF-CD30-IL2R-BLC-TWEAK	Combo CDLXXXVI	0.867	0.875	0.844	0.446
MCP3-S100B-TNFR2-CD30-IL2R-TWEAK	Combo CDXII	0.867	0.844	0.812	0.45
MCP3-S100B-IL2R-TWEAK	Combo CCXXXVI	0.867	0.906	0.75	0.344
MCP3-S100B-TNFR2-MIF-CD30-TWEAK	Combo CCCLXXXI	0.867	0.844	0.844	0.503
MCP3-S100B-TNFR2-CD30-IL2R-BLC	Combo CDX	0.867	0.875	0.812	0.433
MCP3-S100B-TNFR2-CD30IL2R	Combo CCLXXXV	0.867	0.844	0.812	0.445
MCP3-S100B-TNFR2-CD30-TWEAK-Eotaxin	Combo CDXVII	0.866	0.781	0.844	0.486
MCP3-S100B-TNFR2-MIF-TWEAK-Eotaxin	Combo CDII	0.866	0.781	0.812	0.559
MCP3-S100B-TNFR2-IL2R-BLC-Eotaxin	Combo CDXXV	0.866	0.719	0.906	0.628
MCP3-S100B-CD30-IL2R-Eotaxin	Combo CCCLVII	0.866	0.906	0.688	0.332
MCP3-S100B-TNFR2-IL2R-Eotaxin	Combo CCCVII	0.866	0.719	0.938	0.663
MCP3-S100B-CD30-IL2R-BLC-TWEAK	Combo CDLI	0.866	0.906	0.781	0.364
MCP3-S100B-IL2R-BLC-TWEAK	Combo CCCLXVIII	0.866	0.875	0.781	0.406
MCP3-S100B-TNFR2-MIF-IL2R-TWEAK	Combo CCCXCVII	0.866	0.781	0.875	0.549
MCP3-S100B-TNFR2-MIF-CD30	Combo CCLI	0.866	0.844	0.844	0.507
MCP3-S100B-IL2R-BLC-Eotaxin	Combo CCCLXIX	0.865	0.719	0.906	0.621
MCP3-S100B-TNFR2-CD30-IL2R-Eotaxin	Combo CDXIII	0.865	0.906	0.719	0.355
MCP3-S100B-TNFR2-CD30-BLC-Eotaxin	Combo CDXVI	0.864	0.75	0.875	0.648
MCP3-S100B-TNFR2-CD30-Eotaxin	Combo CCXCI	0.864	0.75	0.875	0.648
MCP3-S100B-TNFR2-MIF-Eotaxin	Combo CCLXXI	0.864	0.781	0.812	0.568
MCP3-S100B-TNFR2-MIF-IL2R-BLC-TWEAK	Combo CDLXXIII	0.864	0.812	0.875	0.531
MCP3-S100B-CD30-BLC-TWEAK	Combo CCCLIX	0.864	0.906	0.812	0.463
MCP3-S100B-MIF-CD30-BLC-TWEAK-Eotaxin	Combo CDLXXXIX	0.863	0.781	0.844	0.509
MCP3-S100B-TNFR2-CD30-BLC-TWEAK-Eotaxin	Combo CDLXXXII	0.863	0.781	0.844	0.506
MCP3-S100B-TNFR2-MIF-BLC-TWEAK-Eotaxin	Combo CDLXXVI	0.863	0.75	0.844	0.563
MCP3-S100B-TNFR2-MIF-CD30-TWEAK-Eotaxin	Combo CDLXVI	0.863	0.875	0.75	0.382
MCP3-S100B-MIF-CD30-TWEAK-Eotaxin	Combo CDXXXVIII	0.863	0.781	0.844	0.512
MCP3-S100B-CD30-TWEAK-Eotaxin	Combo CCCLXI	0.863	0.781	0.844	0.499
MCP3-S100B-TWEAK-Eotaxin	Combo CCXLI	0.863	0.75	0.875	0.599
MCP3-S100B-MIF-CD30-BLC-Eotaxin	Combo CDXXXVII	0.863	0.781	0.844	0.5

MCP3-S100B-TNFR2-MIF-CD30-Eotaxin	Combo CCCLXXXII	0.863	0.875	0.75	0.388
MCP3-S100B-TNFR2-CD30-BLC-TWEAK	Combo CDXV	0.863	0.844	0.875	0.518
MCP3-S100B-CD30-BLC-TWEAK-Eotaxin	Combo CDLIV	0.862	0.781	0.844	0.502
MCP3-S100B-MIF-BLC-TWEAK-Eotaxin	Combo CDXLVIII	0.862	0.75	0.906	0.637
MCP3-S100B-TNFR2-MIF-CD30-BLC-Eotaxin	Combo CDLXV	0.862	0.875	0.75	0.39
MCP3-S100B-CD30-BLC-Eotaxin	Combo CCCLX	0.862	0.781	0.844	0.491
MCP3-S100B-TNFR2-MIF-BLC-Eotaxin	Combo CDI	0.862	0.75	0.844	0.575
MCP3-S100B-BLC-Eotaxin	Combo CCXL	0.862	0.75	0.906	0.648
MCP3-S100B-MIF-CD30-Eotaxin	Combo CCCXXVI	0.862	0.812	0.812	0.477
MCP3-S100B-MIF-CD30-BLC-TWEAK	Combo CDXXXVI	0.862	0.875	0.844	0.48
MCP3-S100B-MIF-CD30-IL2R-TWEAK	Combo CDXXXIII	0.862	0.844	0.844	0.487
MCP3-S100B-CD30-IL2R-TWEAK	Combo CCCLVI	0.862	0.844	0.844	0.487
MCP3-S100B-MIF-CD30-IL2R-BLC	Combo CDXXXI	0.862	0.875	0.781	0.41
MCP3-S100B-TNFR2-MIF-IL2R-BLC	Combo CCCXCV	0.862	0.781	0.844	0.569
MCP3-S100B-MIF-CD30IL2R	Combo CCCXX	0.862	0.875	0.781	0.423
MCP3-S100B-TNFR2-BLC-TWEAK-Eotaxin	Combo CDXXVII	0.861	0.75	0.906	0.647
MCP3-S100B-CD30-Eotaxin	Combo CCXXI	0.861	0.781	0.844	0.515
MCP3-S100B-TNFR2-Eotaxin	Combo CLXVI	0.861	0.75	0.906	0.649
MCP3-S100B-TNFR2-MIF-IL2R	Combo CCLXV	0.861	0.812	0.812	0.5
MCP3-S100B-BLC-TWEAK-Eotaxin	Combo CCCLXXI	0.86	0.75	0.875	0.617
MCP3-S100B-MIF-BLC-Eotaxin	Combo CCCXLV	0.86	0.75	0.906	0.649
MCP3-S100B-TNFR2-IL2R-BLC	Combo CCCIV	0.86	0.781	0.875	0.618
MCP3-S100B-CD30-IL2R	Combo CCXV	0.86	0.875	0.781	0.422
MCP3-S100B-TNFR2-MIF-CD30-BLC-TWEAK-Eotaxin	Combo CDXCVII	0.859	0.875	0.75	0.386
MCP3-S100B-MIF-TWEAK-Eotaxin	Combo CCCXLVI	0.859	0.75	0.875	0.611
MCP3-S100B-TNFR2-TWEAK-Eotaxin	Combo CCCXI	0.859	0.75	0.906	0.648
MCP3-S100B-TNFR2-BLC-Eotaxin	Combo CCCX	0.859	0.75	0.906	0.648
MCP3-S100B-TNFR2-MIF-BLC-TWEAK	Combo CD	0.859	0.844	0.812	0.527
MCP3-S100B-TNFR2-IL2R	Combo CLX	0.859	0.781	0.875	0.62
MCP3-S100B-CD30-IL2R-BLC	Combo CCCLIV	0.858	0.875	0.781	0.409
MCP3-S100B-TNFR2-MIF-CD30-IL2R	Combo CCCLXXIV	0.858	0.875	0.875	0.472
MCP3-S100B-MIF-Eotaxin	Combo CCI	0.857	0.75	0.875	0.626
MCP3-S100B-TNFR2-BLC-TWEAK	Combo CCCIX	0.857	0.812	0.875	0.602
MCP3-S100B-TNFR2-TWEAK	Combo CLXV	0.857	0.812	0.844	0.577
MCP3-S100B-TNFR2-CD30-TWEAK	Combo CCXC	0.856	0.812	0.875	0.579
MCP3-S100B-CD30-TWEAK	Combo CCXX	0.856	0.875	0.781	0.476
MCP3-S100B-Eotaxin	Combo CX	0.855	0.781	0.844	0.57
MCP3-S100B-TNFR2-MIF-TWEAK	Combo CCLXX	0.855	0.844	0.781	0.5

MCP3-S100B-MIF-CD30-TWEAK	Combo CCCXXV	0.854	0.812	0.844	0.534
MCP3-S100B-TNFR2-CD30-BLC	Combo CCLXXXVIII	0.854	0.781	0.875	0.564
MCP3-S100B-MIF-IL2R	Combo CXCV	0.854	0.812	0.781	0.451
MCP3-S100B-TNFR2CD30	Combo CXLVI	0.854	0.812	0.844	0.567
MCP3-S100B-TNFR2-MIF	Combo CXXXI	0.854	0.875	0.781	0.503
MCP3-S100B-TNFR2	Combo LIV	0.853	0.812	0.812	0.579
MCP3-S100B-IL2R-BLC	Combo CCXXXIV	0.852	0.875	0.719	0.327
MCP3-S100B-MIF-CD30-BLC	Combo CCCXXIII	0.852	0.875	0.812	0.491
MCP3-S100B-CD30	Combo XC	0.851	0.875	0.812	0.513
MCP3-S100B-CD30-BLC	Combo CCXVIII	0.849	0.875	0.812	0.508
MCP3-S100B-TNFR2-MIF-BLC	Combo CCLXVIII	0.849	0.812	0.812	0.563
MCP3-S100B-MIFCD30	Combo CLXXXI	0.849	0.875	0.75	0.435
MCP3-S100B-TNFR2-BLC	Combo CLXIII	0.847	0.812	0.812	0.59
MCP3-S100B-IL2R	Combo CIV	0.846	0.844	0.781	0.509
MCP3-S100B-MIF-BLC-TWEAK	Combo CCCXLIV	0.84	0.844	0.75	0.484
MCP3-S100B-MIF-TWEAK	Combo CC	0.84	0.844	0.75	0.493
MCP3-S100B-MIF-IL2R-BLC	Combo CCCXXIX	0.84	0.875	0.719	0.407
MCP3-S100B-MIF-BLC	Combo CXCVIII	0.839	0.812	0.75	0.497
MCP3-S100B-MIF	Combo LXXV	0.834	0.812	0.75	0.505
MCP3-S100B-BLC-TWEAK	Combo CCXXXIX	0.829	0.875	0.75	0.478
MCP3-S100B-TWEAK	Combo CIX	0.827	0.906	0.688	0.399
MCP3-S100B-BLC	Combo CVII	0.803	0.938	0.625	0.346
MCP3-S100B	Combo XXVI	0.792	0.906	0.719	0.476

9-biomarker panel (combo DII) is in bold text. Abbreviations: AUC, area under curve; Opt, optimal; SE, sensitivity; SP, specificity.

Table S5. Combinatorial analysis of candidate biomarkers (logistic regression analysis)

Biomarker	AUC	SE	95% CI	The significance level of AUC difference compared to the 9-panel AUC
**Discovery cohort**				
TNF-R2	0.943	0.0309	0.835 to 0.989	P = 0.0651
MIF	0.931	0.0396	0.819 to 0.984	P = 0.0814
CD30	0.872	0.0525	0.743 to 0.951	**P = 0.0148**
MCP-3	0.862	0.0602	0.732 to 0.944	**P = 0.0219**
IL-2R	0.84	0.0595	0.706 to 0.930	**P = 0.0072**
BLC	0.836	0.0602	0.701 to 0.927	**P = 0.0064**
TWEAK	0.786	0.0665	0.644 to 0.891	**P = 0.0013**
Eotaxin	0.752	0.0717	0.606 to 0.865	**P = 0.0005**
S100B	0.729	0.074	0.582 to 0.847	**P = 0.0003**
9-biomarker panel (MCP3/S100B/TNFR2/MIF/CD30 /IL2R/BLC/TWEAK/Eotaxin)	1	0	0.926 to1.000	
**Validation cohort**				
TNF-R2	0.814	0.0554	0.698 to 0.901	P = 0.2739
MIF	0.786	0.0598	0.666 to 0.879	P = 0.1544
CD30	0.782	0.0583	0.661 to 0.876	P = 0.1325
MCP-3	0.776	0.0588	0.655 to 0.871	P = 0.1144
IL-2R	0.771	0.0622	0.648 to 0.866	P = 0.1126
BLC	0.762	0.0598	0.639 to 0.859	P = 0.0796
TWEAK	0.733	0.0628	0.608 to 0.836	**P = 0.0376**
Eotaxin	0.717	0.0659	0.590 to 0.822	**P = 0.0267**
S100B	0.697	0.067	0.570 to 0.806	**P = 0.0146**
9-biomarker panel (MCP3/S100B/TNFR2/MIF/CD30 /IL2R/BLC/TWEAK/Eotaxin)	0.89	0.0419	0.786 to 0.954	

Significant differences between 9-panel AUC and individual biomarker AUC are in bold text.
Abbreviations: AUC, area under curve; CI, confidence interval; SE, standard error.
